# Thiogenistein—Antioxidant Chemistry, Antitumor Activity, and Structure Elucidation of New Oxidation Products

**DOI:** 10.3390/ijms23147816

**Published:** 2022-07-15

**Authors:** Elżbieta U. Stolarczyk, Weronika Strzempek, Marta Łaszcz, Andrzej Leś, Elżbieta Menaszek, Krzysztof Stolarczyk

**Affiliations:** 1 Analytical Department, Łukasiewicz Research Network–Industrial Chemistry Institute, 8 Rydygiera Street, 01-793 Warsaw, Poland; elzbieta.stolarczyk@ichp.pl (E.U.S.); m.laszcz@nil.gov.pl (M.Ł.); 2 Faculty of Chemistry, Jagiellonian University, 2 Gronostajowa Str., 30-387 Krakow, Poland; weronika.strzempek@doctoral.uj.edu.pl; 3 Faculty of Pharmacy, Collegium Medicum, Jagiellonian University, 9 Medyczna Str., 30-068 Krakow, Poland; elzbieta.menaszek@uj.edu.pl; 4 Department of Falsified Medicines and Medical Devices, National Medicines Institute, Chełmska 30/34, 00-725 Warsaw, Poland; 5 Faculty of Chemistry, University of Warsaw, 1 Pasteura Street, 02-093 Warsaw, Poland; ales@chem.uw.edu.pl

**Keywords:** antioxidant, antitumor, biologically active compounds, electrochemistry, electrospray ionization, structure elucidation, identification, molecular modeling, oxidation mechanisms, spectroscopic data

## Abstract

Isoflavonoids such as genistein (GE) are well known antioxidants. The predictive biological activity of structurally new compounds such as thiogenistein (TGE)–a new analogue of GE–becomes an interesting way to design new drug candidates with promising properties. Two oxidation strategies were used to characterize TGE oxidation products: the first in solution and the second on the 2D surface of the Au electrode as a self-assembling TGE monolayer. The structure elucidation of products generated by different oxidation strategies was performed. The electrospray ionization mass spectrometry (ESI-MS) was used for identifying the product of electrochemical and hydrogen peroxide oxidation in the solution. Fourier transform infrared spectroscopy (FT-IR) with the ATR mode was used to identify a product after hydrogen peroxide treatment of TGE on the 2D surface. The density functional theory was used to support the experimental results for the estimation of antioxidant activity of TGE as well as for the molecular modeling of oxidation products. The biological studies were performed simultaneously to assess the suitability of TGE for antioxidant and antitumor properties. It was found that TGE was characterized by a high cytotoxic activity toward human breast cancer cells. The research was also carried out on mice macrophages, disclosing that TGE neutralized the production of the LPS-induced reactive oxygen species (ROS) and exhibits ABTS (2,2′-azino-bis-3-(ethylbenzothiazoline-6-sulphonic acid) radical scavenging ability. In the presented study, we identified the main oxidation products of TGE generated under different environmental conditions. The electroactive centers of TGE were identified and its oxidation mechanisms were proposed. TGE redox properties can be related to its various pharmacological activities. Our new thiolated analogue of genistein neutralizes the LPS-induced ROS production better than GE. Additionally, TGE shows a high cytotoxic activity against human breast cancer cells. The viability of MCF-7 (estrogen-positive cells) drops two times after a 72-h incubation with 12.5 μM TGE (viability 53.86%) compared to genistein (viability 94.46%).

## 1. Introduction

Reactive oxygen species (ROS) and reactive nitrogen species (RNS) are the main sources of oxidative stress in biological systems. ROS and RNS can react with proteins, lipids, and nucleic acids, giving rise to damage at various sites within the cell [1]. The cell contains various enzymes and antioxidants (AOXs) to provide protection and avoid damage. AOXs are compounds that significantly delay or inhibit oxidation of the oxidisable substrate at relatively low concentrations [2]. Flavonoids and isoflavonoids such as genistein (GE) are well-known antioxidants and can help protect cells against reducing carcinogenesis. Flavonoids exhibit a broad spectrum of biological activity, including antioxidant, antitumor, antibacterial, antiviral, anti-inflammatory, anti-allergenic, and vasodilatory actions [3,4,5,6,7,8]. Additionally, they play an important role in the prevention and treatment of hormone-dependent diseases. Genistein is thought to act as an anticancer agent in a large part through its ability to scavenge oxidants involved in carcinogenesis. However, the clinical and therapeutic use of genistein still suffers from many problems related mainly to low lipid and water solubility [6] and poor bioavailability [7]. Therefore, a lot of studies have focused on obtaining genistein derivatives (mainly modified by glycosylation, alkylation, esterification, and hydroxylation) and their drug delivery systems that achieved the required pharmacological activity but also showed fewer side effects and therapeutic limitations. Thiolated genistein (TGE), described by Sidoryk and co-workers [8], is composed of the genistein residue bound at the 7-OH site to the hydroxyethyl linker and thioglycolic acid residue. Such a new construction of genistein has several interesting properties. The predictive biological activity of structurally new compounds such as thiogenistein–a new analogue of genistein–becomes an interesting way of designing new drug candidates with promising properties.

The antioxidant activity of flavonoids attracted attention because they cannot only scavenge free radicals, but also reduce free radical formation [9]. The antioxidant activity of flavonoids is primarily exerted by phenolic hydroxyl groups. Interestingly, in certain flavonoids, bond dissociation of the C−H bonds located at C3 of the C-ring competes with the O-H bond, suggesting that dissociation of the C-H bond can be involved in antioxidant activity [9]. The chemical structure plays a fundamental role in the antioxidant activity of substances. It is known that the existence of a certain hydroxylation pattern, particularly, in the B-ring of the flavonoid structure and/or a C2=C3 double bond in conjugation with a C4-carbonyl group, O3−H groups, and methoxyl groups, increases antioxidant activities [10]. The computed here molecular structure of TGE exhibits several properties supposed to shed more light on the molecular basis of this new compound’s antioxidant activity. They possess the ability for effective radical scavenging, an important (substituted) phenolic ring (B), it has a C-2,3 double bond in conjugation with a 4-oxo function in the C-ring. A rather minor influence of the C-ring on electron delocalization from the B-ring is expected. In the TGE molecule, the C and B rings are twisted relative to each other (about 40 degrees). Moreover, the length of the C3-C1’ bond between them is closer to be single (0.149 nm vs. 0.151 nm for single C-C) than double (0.140 nm, as in the B ring). Some of these properties can be recognized in flavonoids (e.g., apigenin) and isoflavonoids (e.g., genistein and daidzein). It is very challenging to correlate the molecular characteristics to antioxidant activity, e.g., [11,12]. Previously reported studies on polyphenolic flavonoid structures have shown that their radical scavenging activity is related to the presence of phenolic hydroxyl groups, through their H-donating properties acting via the hydrogen atom transfer (HAT) or the single electron transfer followed by proton transfer (SETPT) mechanisms [13,14]. It is known that compounds that possess strong radical scavenging abilities are oxidized at lower potentials [15,16]. However, the flaw in this way of thinking is the possible autoxidation of strong radical-scavenging compounds, so their pro-oxidant properties. A balance is needed between antioxidant and pro-oxidant activities. Specific products are formed during these antioxidant reactions. The knowledge of the oxidation mechanisms is crucial to ascertain the influence of the redox behavior on the pharmacological, nutritional, and chemical properties of our new thiolated analogue of genistein and for predicting the mechanism of metabolism.

The aim of the current study was to apply different strategies for the generation of oxidation products of TGE in solution and on 2D surfaces and the subsequent identification of the unknown products. For the 2D oxidation study, TGE was chemically attached to the gold surfaces as the self-assembled monolayers (SAMs). TGE in SAMs has limited conformational space, it is more susceptible for intermolecular interactions, as well as for the oxidation agent attacking various TGE molecular fragments. A multitude of experimental approaches is needed for the characterization of SAMs after oxidation. Attenuated total reflectance spectroscopy (ATR) is a variant of infrared spectroscopy (IR) and is frequently employed to investigate SAMs [17,18]. The multi-tool analytical approach was based on electrochemistry (EC) and mass spectrometry with electrospray ionization (ESI-MS) for the TGE solution oxidation study. The EC was coupled directly to ESI-MS. TGE oxidation products obtained by using electrochemical potentials and reactions with hydrogen peroxide were identified. The use of electrochemical potentials and H_2_O_2_ will model the action of oxidative agents on TGE in various environments. This helps to predict a possible pattern of TGE metabolism. Both physicochemical methods are precisely controlled and have great potential as fast alternatives to in vitro assays. The electroactive centers of TGE are identified and their oxidation mechanisms are determined. It is worth mentioning here that electrochemical techniques have also been applied for showing similarities between electrochemical and biochemical reactions with the use of the structure–activity relationship (SAR) for flavonoids [16,19] possessing redox activity. The molecular modeling and the quantum mechanical density functional calculations are also performed on model systems to support a discussion.

The anticancer and antioxidative activity of the new analogue is evaluated based on cell culture, the radical scavenging activity (ABTS assay), and the generated ROS level (DCFH-DA assay). The research is carried out on one human breast cancer (MCF-7 estrogen positive and MDA-MB-231 estrogen negative cells line) and also on LPS-induced mice macrophages (RAW 264.7 line).

To summarize, this work becomes a continuation of our former study on physicochemical, biological, and theoretical properties of a new compound, TGE [20]. Here, we estimated the oxidation with electrochemical and chemical (H_2_O_2_) methods by identifying the main oxidation products with MS/MS spectrometry and IR spectroscopy, antitumor and antioxidant properties by biological methods, and by theoretical modeling predicting antioxidant activity. It was tested whether the modification of the structure, by introducing an HS-CH_2_-COO-CH_2_-CH_2_- group, would produce an analogue with a higher antitumor and antioxidant potential.

The study of the effect of TGE chemistry on its biological activity is an important aspect. We hope that understanding these effects will help to determine key molecular fragments involved in the subsequent biochemical processes in living organisms and extend information about their role in metabolic processes and pharmacological activity [21].

## 2. Results and Discussion

Genistein is one of the isoflavones with extensively studied antioxidant and antitumor potentials. It modulates various steps of the cell cycle, angiogenesis, apoptosis, and metastasis in different types of cancers [22,23]. Therefore, the number of studies on the synthesis and biological evaluation of new genistein derivatives is increasing every year. In our research, we focused on a new thioderivative of genistein and its biological and antioxidant properties.

### 2.1. ABTS Radical ScavengingAssay

**Figure 1:**
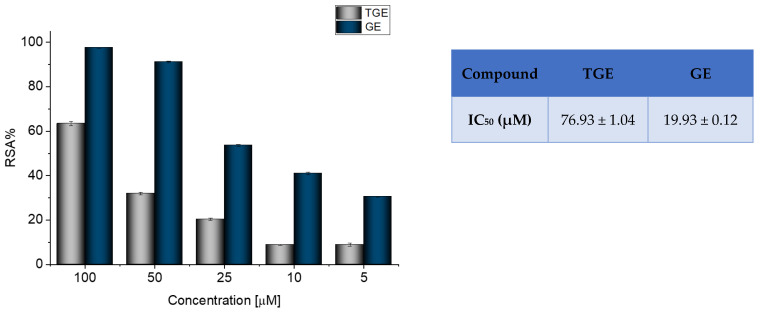
Determination of the antioxidant activity of GE and TGE by ABTS radical scavenging assay. The data shown were obtained after 30 min of incubation. The value of IC_50_ (half-maximal inhibitory concentration) in the figure inset is represented as mean ± SD (n = 3).

Radical scavenging activity (RSA%) measured by ABTS correlates with the electron or hydrogen donating ability of antioxidants, and it is the mainly used method to evaluate the antioxidant activity of studied compounds. The inhibitory effects of TGE and GE on ABTS radicals are shown in Figure 1.

The previously described studies of the flavonoids polyphenolic structures have shown that hydroxyl groups and their structural arrangements induce antioxidant properties through the H-donating transfer. After 30 min of incubation, both TGE and GE possessed inhibitory effects on the ABTS radical in a dose-dependent manner. However, the test performed for TGE (4′,5-hydroxyisoflavone) versus GE (4′,5,7-dihydroxyisoflavone) confirmed that conversion of the 7-hydroxy structure in the A ring of GE to the thiolated residue in the A ring of TGE reduced the value of RSA% TGE to 64.97%, compared to 97.35% GE. The IC_50_ values of TGE and GE were 76.93 (±1.04) and 19.93 (±0.12) μM, respectively.

### 2.2. H_2_DCF-DA ROS Detection Assay

**Figure 2:**
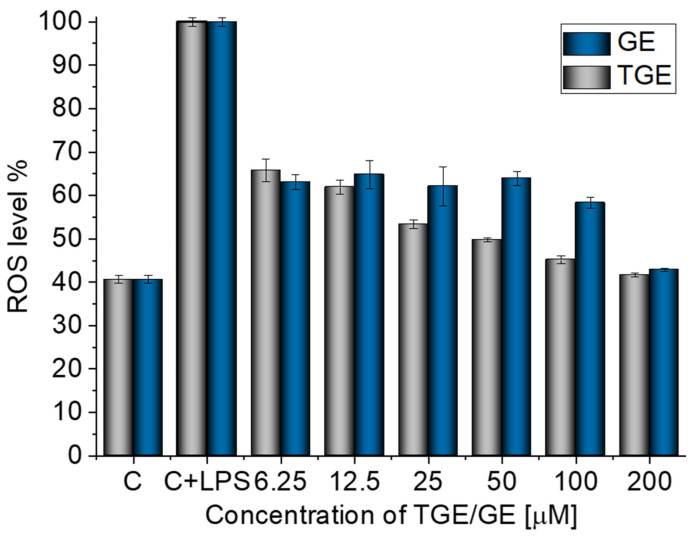
Effective inhibition of LPS-induced ROS production by TGE and GE solutions in macrophage cells after 24 h of incubation (C+LPS means cells stimulated by LPS and C means non-stimulated cells/control group).

The inhibition activity of LPS-induced ROS production by TGE at different concentrations in macrophage cells was determined by using the DCFH-DA assay. The LPS-treated (10 µg/mL) macrophages showed an increase in reactive oxygen species production of approximately 60% more ROS compared to non-stimulated cells (Figure 2). The addition of TGE and GE solutions in the tested range significantly reduced the LPS-induced ROS production in RAW 264.7 cells. In the case of cells treated with the two highest concentrations of TGE, 200 and 100 µM observed an approximate 58.29% (±0.40%) and 54.82% (±0.93%) reduction, respectively. The level of ROS was similar to those generated by non-stimulated macrophages. During the concentration of 6.25 µM, the effect for TGE and GE is similar. Compared to the LPS-induced cells, the ROS level is decreased by 34.2% (±2.58%) and 36.94% (±1.74%) for TGE and GE, respectively.

The presence of the -SH group in the new substituent may be responsible for the correct thiol–disulfide balance and the associated oxidation–reduction potential of cells. A similar mechanism is observed for glutathione, which is a natural antioxidant [20,24].

### 2.3. In Vitro Study–Cell Viability, Cytotoxicity

Studies on synthetic genistein derivatives, including combinations with glycosides, for example, prove the effectiveness of the anti-tumor activity in in vitro systems. The potency of the anti-tumor activity of genistein glycosides varies depending on the sugar groups attached. For example, the addition of acetylated sugar hydroxyls to genistein resulted in a greater selectivity for cancer cells. Additionally, it was confirmed that the potency of the antitumor activity of genistein and its derivatives varies in different tumor types, depending on their selectivity for the target molecules. Our previous studies have shown that the substitution of the -OH group at position seven of the A ring by an ethyl linker and a thioglycolic acid residue allows us to obtain a derivative showing a higher cytotoxic activity against human prostate cancer DU145 cells, but also a lower toxicity against normal prostate epithelial cells (PNT2) compared to its precursor–genistein [20]. Based on previous promising results, our research has been extended to include two breast cancer lines: the estrogen-positive MCF-7 and the estrogen-negative MDA-MB-231.

#### Antitumor Activity–Breast Cancer

Breast cancer belongs to the group of heterogeneous diseases with many clinical, molecular, and histopathological forms, which makes obtaining effective chemotherapy problematic. Seventy percent of them are associated with the expression of the estrogen-α (ERα) receptor. Therefore, as a model to determine the anti-tumor activity of TGE, two breast cancer lines, MCF-7, an estrogen-positive breast cancer, and MDA-MB-231, an estrogen-negative breast cancer, are used.

**Figure 3:**
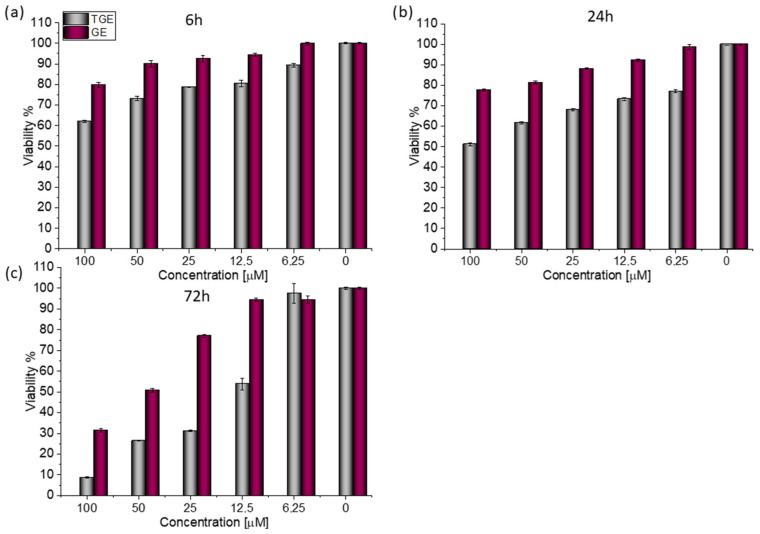
The viability of MCF-7 cells treated by different concentrations of GE and TGE after (**a**) 6 h, (**b**) 24 h, and (**c**) 72 h (determined by PrestoBlue^TM^ test). Presented data are representative of two independent experiments and are expressed as the mean ± SD. The error bars represent the ±SD.

For both lines, similar relationships were obtained, as in the case of prostate cancer [20]. After 72 h (Figure 3c), the viability drops to 8.76% (±0.20%) for TGE. For the sake of comparison, the administration of genistein reduced this parameter only to 31.42% (±0.73%). The use of the lower concentration of 12.5 µM TGE is sufficient to reduce the viability of the cells to 53.86% (±2.79%). When the same concentration of GE is administered, there is no effect visible in the reduction of viability. This indicates that TGE is more effective in inhibiting the growth of these ERα-positive breast cancer cells at much lower concentrations than genistein.

**Figure 4:**
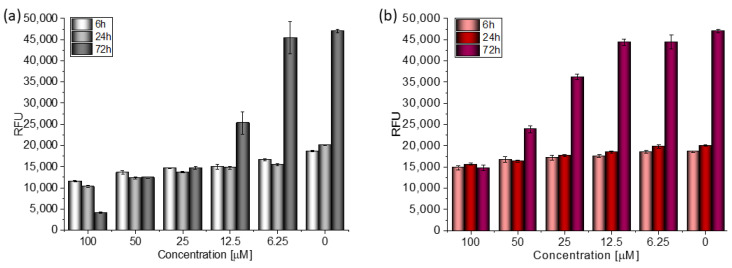
The proliferation rate of MCF-7 cells correlated with relative fluorescence units (RFU) obtained from all cells in the samples with PrestoBlue^TM^ test after 6 h, 24 h, and 72 h of incubation with (**a**) TGE and (**b**) GE. Untreated cells were used as references. The obtained results are proportional to the number of cells. The data are representative of two independent experiments and are expressed as the mean ± SD. The error bars represent the ±SD.

Based on the value of the relative fluorescence intensity (RFU) recorded after 6, 24, and 72 h of incubation of MCF-7 cells with 100 µM TGE, not only an arrest of proliferation was observed, but also a reduction in the number of living cells, which may indicate their death (Figure 4). This effect was also observed for the highest (100 μM) concentration of TGE, while it was not visible for pure GE. Note that genistein is one of the major soy isoflavones and exhibits a relative nontoxicity. However, the structural similarity of genistein with 17β-estradiol, means that GE can bind to Erα+ receptors and induce both agonistic and antagonistic effects on MCF-7 cells proliferation. Thus, at a lower dose, GE may stimulate ERα+ cell growth and entry into the cell cycle [25,26]. In the present study, following treatment for three points of time, TGE significantly inhibited the proliferation of MCF-7 cells in a concentration-dependent manner, may offset effects related to a biphasic effect, and could be beneficial for breast cancer even in lower concentrations.

**Figure 5:**
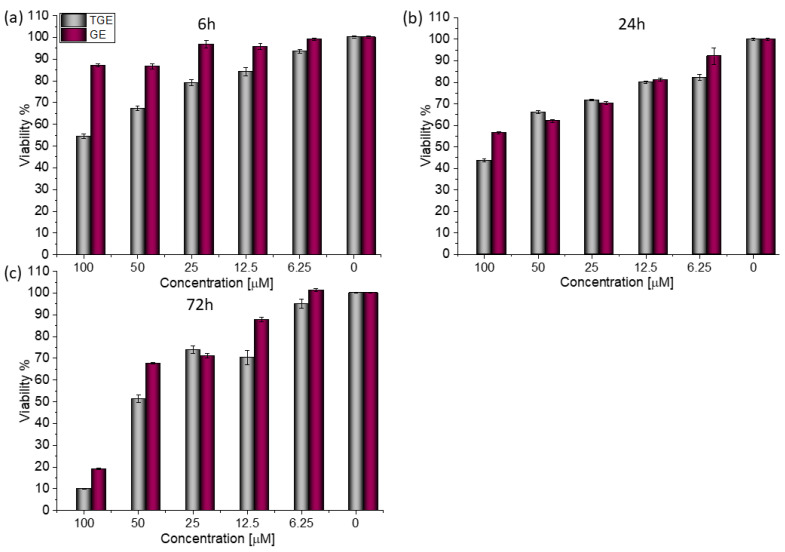
The viability of MDA-MB-231 cells treated by different concentrations of GE and TGE after (**a**) 6 h, (**b**) 24 h, and (**c**) 72 h (determined by PrestoBlue^TM^ test). Presented data are representative of two independent experiments and are expressed as the mean ± SD. The error bars represent the ±SD.

In the case of the MDA-MB-231 breast cancer line (estrogen-negative neoplasm), antitumor activity is also observed after the administration of TGE (Figure 5).

**Figure 6:**
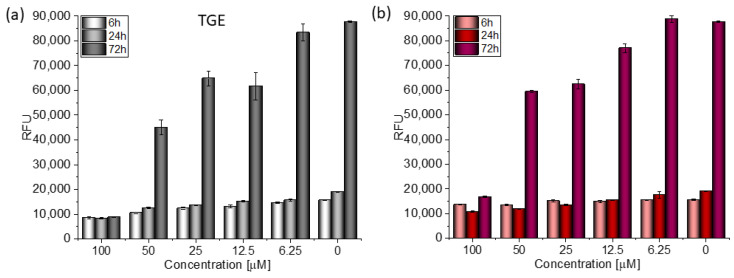
The proliferation rate of MDA-MB-231 cells correlated with relative fluorescence units (RFU) obtained from all cells in the samples with PrestoBlue^TM^ test after 6 h, 24 h, and 72 h of incubation with (**a**) TGE and (**b**) GE. Untreated cells were used as references. The obtained results are proportional to the number of cells. The data are representative of two independent experiments and are expressed as the mean ± SD. The error bars represent the ±SD.

After 72 h of incubation with 100 μM solutions, cell viability decreased to 9.99% (±0.05%). For comparison, the viability of cells incubated with the two highest concentrations of GE was 19.12% (±0.21%) and 67.68% (±0.26%) (Figure 6). Based on the available literature, this effect may be associated with the induction of MDA-MB-231 cells apoptosis by genistein and its derivative and inhibition of proliferation by arresting the cell cycle in the phase G2/M [27]. Additionally, the research presented by H. Pan and coworkers reveals that GE could inhibit the activity of NF-κB via the Notch-1, which may be an explanation mechanism of the downregulation of proliferation. Moreover, it was confirmed that GE downregulated the expression of cyclin B1, Bcl-2, and Bcl-xL in MDA-MB-231 cells, possibly mediated by NF-κB activation via the Notch-1 signaling pathway.

In vitro studies would suggest that changing the hydroxyl for an ether containing an –SH group on the C7 carbon in the A ring may increase the cytotoxic properties of TGE. This effect can be attributed to the presence of a highly reactive -SH group [20,28]. Marik and coworkers also proved that the substitution of the -OH group at position seven of the A ring by aliphatic chains and heterocyclic 1,2,3-triazole moieties enhances the cytotoxic properties. Additionally, modifying the structure of genistein by incorporating structural features with bulky and flexible lipophilic substitutions on the genistein scaffold can increase their binding affinities for estrogen α-receptors. These results indicate that the 7-O-substitutes of genistein are an effective approach to obtaining compounds with an improved antiproliferative activity [29].

To sum up, both TGE and GE have the ability to act against neoplastic activity towards estrogen-negative and estrogen-positive cells, which makes them potential substances in the fight against breast cancer. The obtained results constitute the basis for extending the research to include the characteristics of the action of the new derivative both on individual phases of the cell cycle, as well as the induction of biochemical markers and targeted receptor activity.

### 2.4. Antioxidant Chemistry–Oxidation of TGE by Potential and With Hydrogen Peroxide

The ROXY™ electrochemical system is a technique that can be applied for mimicking drug metabolism [30,31] or for interaction studies [32] as well as other electrochemical cells coupled on-line to the electrospray ionisation (ESI)-MS for study of biological redox reactions [33]. In the work of Sagandykova and co-workers [34], the electrochemical unit coupled to ESI-MS was used for fragmentation activity relationships of selected flavonoids. Selected compounds were subjected to electrochemical degradation and the differences in formed products were analysed for correlation with their structures and antioxidant activity.

The TGE itself as a template was used for the interpretation of the unknown structures of products created during the oxidation of TGE. The product ion analysis was obtained and specific product ions and neutral losses were assigned to the substructures of the molecule. To the best of our knowledge, the MS fragmentation of TGE has not been discussed in the literature. The EPI spectrum of pseudomolecular ions displayed a lot of product ions, which are presented in Table 1. The structures of characteristic fragments, which are proposed, are presented in Figure S1. There are many strategies to identify unknown structures. The one used in this article is based on the assumption that much of the parent compound structure will be retained in the new products or decomposition products. The product ion mass spectrum of the parent compound and fragmentation pattern of the parent compound are used as the templates for the identification of the unknown structure. An orthogonal approach was used to identify new compounds. On the one hand, identification is based on the fragmentation spectrum and on the other hand, consideration of the mechanisms (logical oxidation pathways) that generate the formation of a particular structure.

The formation of the TGE electrochemical oxidation product was monitored by ESI-MS in negative polarization and the results are summarised in Table 1. The formation of the TGE degradation product after treatment with hydrogen peroxide is summarised in Table 2. Very interesting results were obtained using two oxidizing agents simultaneously: hydrogen peroxide and potential, which are summarized in Table 3. TGE without being subjected to oxidation was analysed as a controls sample and is subject only to possible transformation in ESI source, which is an electrochemical cell by itself but with constant potential [35]; in this case, 4.5 kV. This data may be also indicative of electrochemical reactions and provide insights into structure–activity studies.

In the TGE mass spectrum recorded for the reference/control sample, the ion at *m/z* 773 is presented. This is a dimer of the TGE molecule formed by bridging as a disulfide. The compound of M = 773 Da was decomposed when two oxidizing agents were applied simultaneously: hydrogen peroxide and potential. In addition, ions at *m/z* 355 and 339 are present in the reference spectrum of TGE. They are probably the result of uncontrolled fragmentation in the ion source and do not appear in the TGE fragmentation spectrum. The ions at *m/z* 355 and 339 are formed by the loss of two oxygen molecules and three oxygen molecules, respectively. The observed ion at m/z 369 is formed by the loss of a water molecule and is a characteristic signal in the TGE fragmentation spectrum. Furthermore, these are ions whose intensity increases during potential oxidation. Additionally, TGE adducts/polymerization with oxidation products are observed, exemplified by *m/z* ions 836, 819, 811, 809, 795, 773, 749, and 673 with low intensities and very complicated mass spectra. Therefore, these spectra were not analyzed.

**Figure 7:**
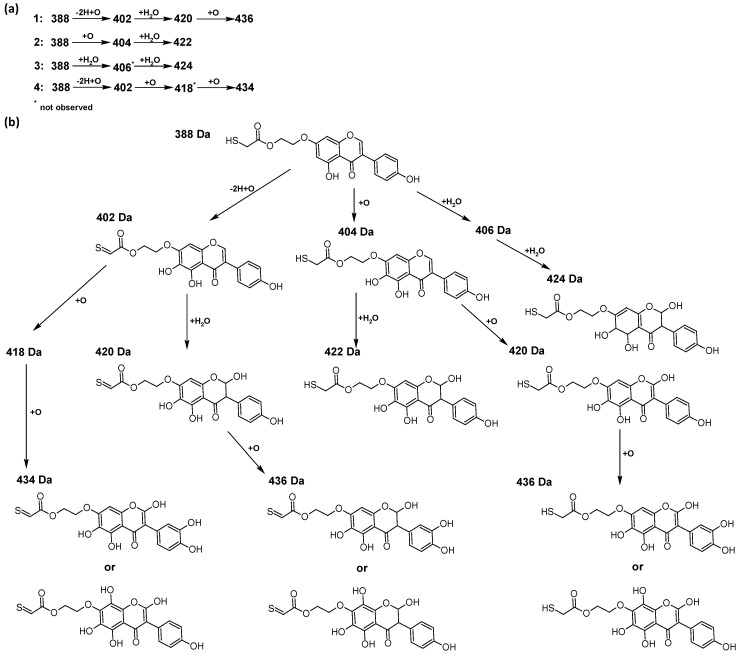
The proposed oxidation pathways of TGE (**a**) and structures of main reaction products (**b**) formed by the oxidation with potential.

**Figure 8:**
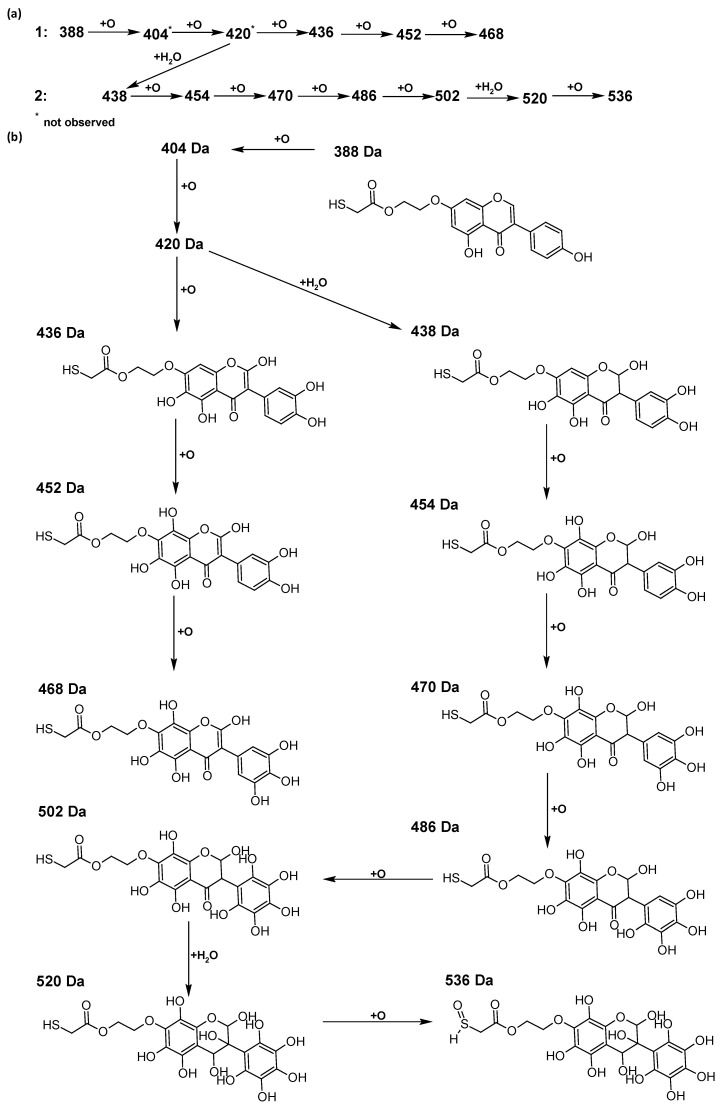
The proposed oxidation pathways of TGE (**a**) and structures of main reaction products (**b**) formed by the oxidation with hydrogen peroxide.

**Figure 9:**
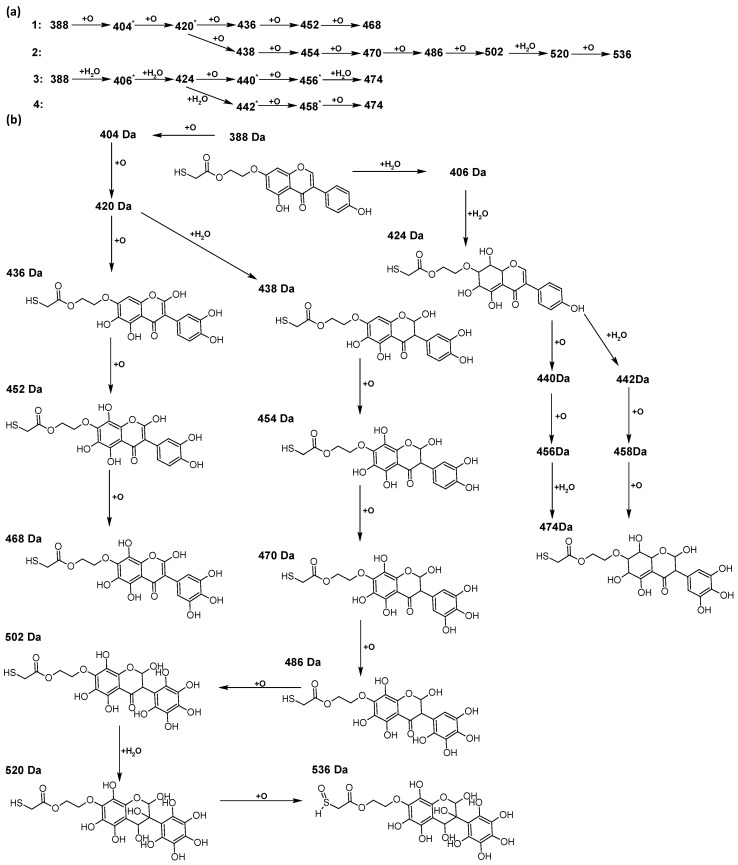
The proposed oxidation pathways of TGE (**a**) and structures of main reaction products (**b**) formed by the oxidation with potential and hydrogen peroxide. * not observed.

In addition to the described background, there are analytically significant signals in the spectra. Based on the fragmentation spectra, structures were proposed for **19** new compounds–oxidation products–in three different environments. Compounds **1**–**7** are characteristic of potential oxidation (Table 1). Compounds **8**–**17** are formed by oxidation with hydrogen peroxide (Table 2). The combination of the two oxidizing agents’ potential and hydrogen peroxide generates the formation of compounds **8**–**17**, the same as in the oxidation with hydrogen peroxide, and in addition, two new compounds **18** and **19** appear (Table 3). The proposed fragmentation pathway of new compounds and structures of characteristic fragments are presented in Figures S2–S15. Based on the proposed TGE potential oxidation products, four oxidation mechanisms are proposed for oxidation with potential (Figure 7a), two different TGE oxidation pathways for oxidation with hydrogen peroxide (Figure 8a) and the same oxidation pathways 1 and 2 as for hydrogen peroxide, and two new pathways 3 and 4 for the two oxidizing factors potential and hydrogen peroxide occurring simultaneously (Figure 9a). The structures for the major compounds according to the proposed oxidation mechanisms are shown in Figure 7, Figure 8 and Figure 9b.

The compounds of M = 404, 406, 418, 420, 440, 442, 456, and 458 Da occur in the proposed oxidation pathways but are not observed in the spectra. These compounds are unstable and are rapidly converted to further structures, so they are seen with low intensities or not observed at all. It is likely that in an active environment these compounds are unstable and undergo further oxidation. However, the formation of compounds that differ by 2Da and that can be combined into characteristic pairs, i.e., 404/406, 418/420, 420/422, 422/424, 434/436, 436/438, 452/454, and 468/470 related to oxidation with the attachment of a water molecule to TGE structure or oxidation alone, was observed. Compounds of M = 402 Da, M = 486 Da, 502 Da, 520 Da, 536 Da, and 474 Da also occupy an important place in the oxidation tracts.

The most intense ion in the MS spectrum of TGE, after potential oxidation, is the ion at *m/z* 402 (compound **1**). The EPI spectrum of this pseudomolecular ion displayed a lot of products ions, which are presented in Table 1. The structures of characteristic fragments, which are proposed, are presented in Figure S2. Based on these data, the structure of the compound **1** has been assigned as 15-thioformyl-6-hydroxy-TGE. During the formation of this compound, dehydrogenation occurs first in the aliphatic side chain (the mildest form of oxidation) and then oxidation in the A ring of TGE. This compound contains an additional -OH group in the A ring in the ortho position. There are ions *m/z* 384, 268 in the fragmentation spectrum that indicate this order. Fragmentation proceeds by elimination of the water molecule from the C ring, therefore two -OH groups are adjacent to each other (the ion at *m/z* 384). Additionally, the ion confirming such an arrangement of -OH groups is fragmented at *m/z* 177. From the fragment at *m/z* 384, the neutral molecule of M = 116 Da (S=CHCOOCHCH_2_) is eliminated, generating a fragment at *m/z* 268, while from the fragment at *m/z* 374, the neutral molecule of M = 132 Da (S=CHCOOCH_2_CHO) is eliminated, generating a fragment at *m/z* 241. These eliminations indicate the occurrence of dehydrogenation at sulfur. It was initially assumed that this compound is formed by oxidation in the C ring; however, a correlation was observed between the compound of M = 402 Da and the compound of M = 420 Da, where the compound of M = 420 Da (compound **3**) is formed by the attachment of a water molecule. Therefore, the compound of M = 402 Da must have a double bond in the C-ring to which a molecular water attaches to form the compound of M = 420 Da. The EPI spectrum of pseudomolecular ions at *m/z* 419 displayed a lot of product ions, which are presented in Table 1. The structures of characteristic fragments, which are proposed, are presented in Figure S3. Based on these data, the structure of compound **3** has been assigned as 15-thioformyl-2,3-dihydro-2,6-dihydroxy-TGE. A mass of 424 Da is formed during both oxidation with potential and oxidation with hydrogen peroxide with potential, but these are two different compounds due to their different fragmentation spectra, the interpretations of which are shown in Figures S4 and S5, respectively. In the fragmentation spectrum of the compound formed during the action of hydrogen peroxide with potential, there is a characteristic fragment *m/z* 339 formed by loss of the neutral moiety of M = 56 Da (O=C=C=O) from the ion at *m/z* 395. Therefore, compound **18** is formed by the attachment of two water molecules to the A ring of TGE. Based on these data, the structure of the compound **18** has been assigned as 6,7,8,9-tetrahydro-6,8-dihydroxy-TGE. In contrast, compound **5** is formed by the attachment of two water molecules, with the first oxidation going to A ring and the second to C ring. Based on these data, the structure of the compound 5 has been assigned as 2,3,6,7-tetrahydro-2,6-dihydroxy-TGE. During oxidation by potential, a compound of M = 436 Da (compound **7**) is formed from compound of 420 Da by oxidation in the ortho position. In this case, the fragmentation spectrum is not clear, and it is not possible to indicate whether this oxidation involves the A or B ring, but it is certainly an oxidation in the ortho position. The rationale behind this interpretation is the proposed structure for the fragment at *m/z* 176. However, it was observed that the neutral molecule S=C=C=O falls off, which confirms the assumption of oxidation by dehydrogenation in the aliphatic part of the TGE. The proposed fragmentation pathway of compound **7** and structures of characteristic fragments, which confirmed the proposed structures, is presented in Figure S6. The product of mass at M = 436 Da (compound **8**) is also formed when hydrogen peroxide and potential with hydrogen peroxide are applied, but it is a different compound due to the different fragmentation spectra. The proposed fragmentation pathway of impurity eight and structures of characteristic fragments, which confirmed the proposed structures, is presented in Figure S7. These compounds are formed by two different oxidation mechanisms. The fragmentation spectrum of compound **8** shows a characteristic fragment at *m/z* 349, which confirms the occurrence of two -OH groups in the B-ring by eliminating the neutral fragment of M = 86 Da. In addition, this elimination shows the occurrence of the -OH group in the C-2 position of TGE. Based on these data, the structure of compound **8** has been assigned as 2,6,3′-trihydroxy-TGE.

Further oxidation of compound **8** with hydrogen peroxide proceeds to carbon C- 8 in the A ring of TGE, generating a compound of mass 452 Da (compound **10**). The proposed fragmentation pathway of compound **10** and the structures of characteristic fragments that confirmed the proposed structure are shown in Figure S8. Based on these data, the structure of compound **10** has been assigned as 2,6,8,3′-tetrahydroxy-TGE. The next oxidation in this sequence proceeds to carbon 5’ in the B ring of TGE, generating the compound of mass 468 Da (compound **12**). The proposed fragmentation pathway of compound **12** and the structures of the characteristic fragments that confirmed the proposed structure are shown in Figure S9. Based on these data, the structure of compound **12** has been assigned as 2,6,8,3′,5′-pentahydroxy-TGE. Compound **12** completes the first oxidation sequence with hydrogen peroxide. In the proposed second sequence of TGE oxidation with hydrogen peroxide, an interesting structure is the compound of M = 520 Da (compound **16**), for which the fragmentation spectrum is characterized by strong signals for ions at *m/z* 474, 457, 435, 430, and 269 (Figure S10). In addition to the saturation of all carbons of A, C, and B rings, additionally in the C ring, an enol is formed from ketone because there is one hydrogen atom in the neighborhood. The interpretation of the fragmentation spectrum for the compound with M = 520 Da shows that the B-ring (saturated with -OH groups) with all -OH groups are very stable. It passes unchanged through all fragments except the ions at *m/z* 435, 406, 390, 178, or *m/z* 122. These are the fragments with the “stripped” B ring. However, it is the *m/z* 435 ion that is the precursor to the *m/z* 390 and 406 ions. The *m/z* 435 ion comes directly from the *m/z* 502 ion because there are no intermediate fragments in the fragmentation spectrum. Based on these data, the structure of the compound **16** has been assigned as 2-hydro-2,3,4,6,8,2′,3′,5′,6′-nonahydroxy-TGE. When it would seem that the oxidation of the TGE molecule with hydrogen peroxide would end with the compound of M = 520 Da, due to the saturation of all carbons, a compound of M = 536 Da appears, as compound **17**. The interpretation of the fragmentation spectrum for compound of M = 536 Da (Figure S11) confirms the presence of oxygen at the sulfur due to several characteristic fragments, e.g., at *m/z* 474, which is formed by elimination of the -OSCH_2_ group, or the *m/z* 454 ion, which is formed by loss of the neutral molecule O=SH-CH_3_. Based on these data, the structure of the compound **17** has been assigned as 16-sulfenic- 2-hydro-2,3,4,6,8,2′,3′,5′,6′-nonahydroxy-TGE.

During the TGE oxidation with hydrogen peroxide and potential simultaneously, an additional oxidation sequence (No. 3, 4) is observed that is not present during the other oxidizing agents. This is a sequence containing a different compound of M = 424 Da (compound **18**) than in the case of oxidation by potential. It is formed by the oxidation of the A ring at positions C-6 and C-8 of TGE as a molecular water addition. The proposed a fragmentation pathway of compound **18** and structures of characteristic fragments, which confirmed the proposed structures, is presented in Figure S5. Based on these data, the structure of compound **18** has been assigned as 6,7,8,9-tetrahydro-6, 8-dihydroxy-TGE. Compound **19** with M = 474 Da completes this oxidation sequence. The suggested fragmentation pathway of compound **19** and the structures of the characteristic fragments that confirmed the proposed structure are shown in Figure S12. Based on these data, the structure of compound **19** has been assigned as 2,3,6,7,8,9-hexahydro-2,6,8,3′,5′-pentahydroxy-TGE. Only in this case, there is an attachment of two water molecules. Oxidation sequence No. 3 and 4 (Figure 9a) are characteristic of the action of hydrogen peroxide and potential simultaneously. Thus, oxidation of the compound alone does not occur. The potential favors the attachment of water molecules.

In summary, three forms of TGE oxidation were observed: the oxidation by water molecule attachment, the oxidation by oxygen attachment, or the dehydrogenation as a mild form of oxidation. The first attack of oxygen probably occurs in the C ring of TGE in the case of oxidation with potential. In addition, the formation of compounds resulting from oxidation on rings with dehydrogenation in the side chain is observed during potential oxidation. Compound **5** is formed by the attachment of two water molecules to the A and C rings. Compounds **3**, **4**, **9**, and **16** are formed by the attachment of a water molecule to the C ring. Compound **18** is formed by the attachment of two water molecules to the A ring. Compound **19** is formed by attaching two water molecules to the A ring and one to the C ring after prior oxidation of the B ring of TGE. The substitution in the A ring is due to an inductive effect. The electrodonor group -OH causes a partial positive charge to appear on the adjacent C atom. The elimination of the neutral moiety -CH_2_O is observed in the fragmentation spectrum. This is a characteristic elimination in fragmentation spectra of compounds that are formed by the oxidation by attachment of a water molecule. It has been observed that the experiment with potential favors the attachment of a water molecule. In this case, a higher number of water molecules are observed in the A ring. In the experiments without the action with potential, there are the water molecules attached mainly to the double bond in the C ring of TGE.

Our experiments are important for predicting the physicochemical properties of the new compound TGE. Electrochemistry, which helps determine metabolism patterns, is in turn considered to have a great potential as a fast alternative to in vitro assays [36]. The prooxidation activity of flavonoids can be directly related to the number of hydroxyl groups in the molecule. Mono- and dihydroxyl flavonoids usually do not show such activity, while in the case of polyhydroxyl compounds (especially when OH groups are attached to the B ring), a significant increase in the production of reactive oxygen species (ROS) is observed. From the present work, it appears that the TGE antioxidant can undergo modifications of the A, C, and B rings upon oxidation. This is a much more extensive structural variation than formerly was thought, that changes occur mainly in the B ring. What is particularly interesting about the results of our product studies with TGE is the variety of antioxidant chemistries displayed by the A ring. TGE produced products that are not typical of well-documented genistein antioxidant reactions: the B ring hydroxylation and formation of radical addition products. We do not observe structures derived from the two significant metabolites of genistein identified by Kullig et al. [37], i.e., 5,7,8,4’-tetrahydroxyisoflavone and 5,7,3’,4’-tetrahydroxyisoflavone, on oxidative metabolism in human liver microsomes. However, it has been reported that GE can be metabolized by the cytochrome P450s to hydroxylated metabolites (6-, 8-, and 3′-hydroxyGEN) [38] and by the gut microbiota to dihydrogenistein (DGEN), 5-hydroxy-equol (5-OH-equol), and 6′-hydroxy-*O*-desmethylangolensin (6′-OH-DMA) [39,40]. We observe that the oxidation by potential proceeds much further (more -OH groups) than was found by Kullig et al. [37] for genistein metabolites in microsomes. In addition, oxidation products are observed that result from dehydrogenation at the -SH group, which are new compounds. The characterization of these product structures provides potential markers of TGE antioxidant reactions. Sensitive assays for specific TGE oxidation products may thus be useful markers for antioxidant reactions of the isoflavone in biological systems.

### 2.5. Description of Qualitative Changes Observed in the IR-ATR Spectra during the Oxidation of the TGE Monolayer on the Au Electrode

The free radical peroxidation of unsaturated lipids in biomembranes disrupts the various important structural functions associated with this natural protective barrier for cells; as a result of this oxidation, various in vivo pathologies can occur [41]. It is known that genistein and its metabolites possess an ability to inhibit lipid peroxidation in biological membranes. It is supposed that molecules are localized near the lipid–water interference of the membrane or/and they are incorporated into its hydrophobic core, which causes an increase in membrane rigidity and stability to the diffusion of free radicals [42]. The aim of our preliminary studies of TGE oxidation on the surface of the Au 2D electrode is to mimic the oxidation of a new GE derivative on the surface of a biomembrane.

#### Vibrational Studies on Oxidative Monolayers–Simulation of Active Substance Immobilization on the Surface of Biological Membranes

At the beginning, it should be emphasized that the following assumption was made regarding the interpretation of IR spectra, that in a tightly packed monolayer, limited by the surface of the electrode, more oxidation products of TGE by H_2_O_2_ are possible due to the differences in the inner and outer environment of the molecule.

**Figure 10:**
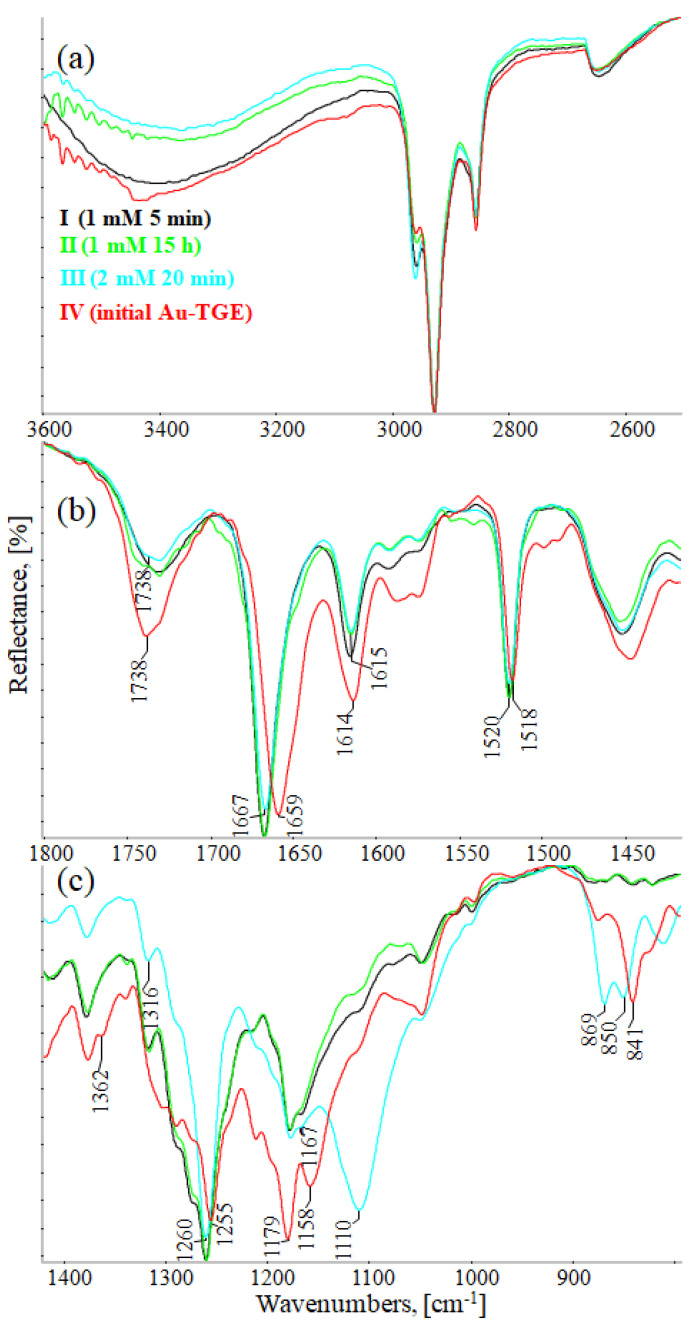
IR-ATR spectra of Au-TGE before and after oxidation with H_2_O_2_, (**a**) from 3600 to 2500 cm^−1^; (**b**) from 1800 to 1425 cm^−1^; (**c**) from 1400 to 800 cm^−1^.

Figure 10a (3600–2500 cm^−1^), 10b (1800–1420 cm^−1^), and 10c (1420–800 cm^−1^) show the comparison of IR-ATR spectra of TGE on the Au electrode before and after oxidation by H_2_O_2_ (spectra: I–1 mM 5 min, II–1 mM 15 h, and III–2 mM 24h). The presence of hydroxyl groups in the TGE molecule before and after oxidation is proven in the IR spectrum by the presence of the broad band between 3600 and 3200 cm^−1^.The presence of a broad band may suggest the absence of hydrogen bonding with hydroxyl groups in the TGE molecule before and after oxidation. At the same time, it should be taken into account that during oxidation more OH groups are added, but in a tightly packed monolayer the outer molecules are much more susceptible to oxidation (which means more hydroxyl groups) than the inner ones because the whole molecule is exposed to H_2_O_2_; for the inner molecules only the B ring will be the most susceptible to oxidation.

Bands from C-H stretching vibrations from the methylene group of the TGE molecule are observed at 2959, 2927, and 2855 cm^−1^. It can be observed that relative intensities, as well as wavenumbers of these bands, remain unchanged during oxidation.

The band from the carbonyl stretching vibration from the chain [20] has the same value of 1738 cm^−1^ before and after oxidation. However, the band from the carbonyl stretching vibration assigned to the ring [20] is shifted towards higher wavenumbers from 1659 to 1667 cm^−1^, which indicates a weakening or breaking of the intramolecular C=O–O-H hydrogen bond. This can be caused by the oxidation of the –OH group in the A ring into a C=O group or by the presence of additional –OH groups as it can be presented in the compounds of M = 436, 452 and 468 Da, as can see in Figure 8.

In IR spectra of TGE, both before and after oxidation, bands from 1615 to 1518 cm^−1^ mainly originate from C=C vibrations. However, their values are slightly shifted towards higher wavenumbers after oxidation, which can indicate changes in aromatic rings during oxidation.

In the range of deformation, vibrations of hydroxyl groups 1400–1300 cm^−1^ following changes that occurred during oxidation are observed: the band at 1362 cm^−1^ disappears and the band at 1316 cm^−1^ rises.

In IR spectra of TGE before oxidation, the doublet at 1179 cm^−1^ and 1158 cm^−1^ originate from C-O-C asymmetric stretching and C-OH stretching vibrations. During oxidation, the band at 1158 cm^−1^ shifts into 1167 cm^−1^ and the new band is formed at 1110 cm^−1^, which probably originates from stretching C-O vibrations and can prove the presence of new –OH groups after oxidation.

In the TGE spectrum before oxidation, the main band from C-H deformation out-of-plane vibrations from aromatic rings is at 841 cm^−1^. During oxidation, bands at 869 cm^−1^ and 850 cm^−1^ are observed that can prove that the oxidation of TGE influences the rings of the molecule.

The comparison of IR spectra does not indicate the polymerization of TGE molecules during oxidation by H_2_O_2_, which is in agreement with the results obtained by Xu and co-workers for the caffeic acid monomer without the presence of horseradish peroxidase [43]. On the other hand, polymerization by Ar-Ar (B rings) is possible but difficult to prove. Likewise, polymerization via Ar-O-Ar (B-rings) is also possible, but the expected aryl ether band is in the range of 1270–1230 cm^−1^ and is found in the spectrum of the initial sample.

From the comparison of the theoretical IR spectra (obtained by the B3LYP calculations) of TGE vs. M436, TGE vs. M452, and TGE vs. M468 (Figures S16–S18), one can see that introducing the OH-groups to the skeleton of the TGE molecule results in the significant modification of the theoretical TGE spectrum in the region of 1300–900 cm^−1^. This is actually the region in the experimental spectrum presented above in Figure 10c as spectrum III (2 mM 20 min), corresponding to the intensive oxidation by H_2_O_2_. However, in this experimental spectrum, only the bands at: 1110, 869, and 850 cm^−1^ have an increased intensity. This qualitative comparison supports our belief that in the 2D area the molecules with additional OH groups in the B ring should dominate.

### 2.6. Molecular Modeling and the Quantum Mechanical Density Functional Calculations

A theoretical estimation of the antioxidant activity of TGE was also studied. There exists an extensive discussion in the literature on how to define the antioxidant power of selected species, starting from colorimetric determination of the total oxidant status (TAS) and the total antioxidant capacity (TAC) [44,45,46]. Here, we apply a theoretical method based on the density functional theory allowing the comparison of various antioxidants using two parameters, i.e., the bond dissociation energy (BDE) and the Gibbs free energy of the antioxidant reaction with H_2_O_2_ [47,48]. As long as many of the antioxidants were investigated experimentally, their antioxidant effectiveness was evaluated, and the above-mentioned parameters were calculated, there is a possibility to evaluate the antioxidant power of new analogues theoretically by a simple comparison. This method allows us to locate TGE within the series or the scale of known antioxidants.

One of the best reliable thermodynamic parameters to describe the hydrogen atom transfer (HAT) mechanism of antioxidant activity is the bond dissociation enthalpy (BDE). A corresponding model reaction is given below:

Ge-OH + R^•^ → Ge-O^•^ + R-H (1)

where Ge-OH denotes, for example, the genistein with the OH group at the C4′ position. R is the radical whose unpaired electron is abstracted by the antioxidant (here the genistein molecule). In this way, the free radical (R^•^) is deactivated by the antioxidant. Such a picture oversimplifies a more complex mechanism because apart from electron transfer the proton is transferred as well.

The bond dissociation enthalpy is defined as follows:

BDE = ***H***(Ge-O^•^) + ***H***(H^•^) − ***H***(Ge-OH) (2)

where ***H*** denotes the enthalpy.

According to this mechanism, the hydroxyl group of the antioxidant compound (Ge-OH) releases the hydrogen atom (H^•^), being transformed to the respective free radical (Ge-O^•^). The higher antioxidant activity corresponds to the weaker O-H bond. In Table 4, the bond dissociation enthalpy (BDE) was estimated with the density functional theory (DFT) and one of its implementations, i.e., the B3LYP/6-311++G(d,p) method for genistein and thiogenistein. For comparison, analogical calculations were carried out for phenol, Trolox, and curcumin. Trolox is a popular antioxidant compound frequently used in the laboratory studies. The BDE was calculated for isolated (non-interacting with environment) molecules.

A series of the BDE values shows that the lowest BDE corresponds to Trolox while the highest BDE can be ascribed to phenol. The BDE values of genistein and thiogenistein are comparable and smaller than the BDE of phenol and curcumin. It suggests that the antioxidant activity of genistein and thiogenistein is governed by the OH-substituted core of these compounds. One can mention that the phenolic residue appears in a plethora of antioxidants [49].

The antioxidant activity of TGE and the related species was estimated also for the reaction with the ABTS^•+^ radical cation (Equation (3)). This is a one electron reduction reaction of the ABTS^•+^ radical cation. A corresponding reaction is given below:

Antioxidant + ABTS^•+^ → Antioxidant^•+^ + ABTS (3)

With the use of the B3LYP/6-31G(d,p) method, the Gibbs free energy output of the Equation (3) reaction was estimated. The corresponding values are presented in the Table 5.

The ΔG results presented in Table 5 may reflect real reactions only qualitatively assuming that the values of Trolox are the reference point and that the ΔG values above 11.44 kcal/mol denote a poorer antioxidant activity than is exhibited by Trolox. From Table 5, one can conclude that the antioxidant activity of the thiolated genistein, i.e., TGE and M26P (genistein substituted at O-7 with a modified thiol residue by the extra -CH_2_- group), seems to be rather poorer than Trolox.

A slight prevalence of the antioxidant activity of TGE over GE predicted with the B3LYP/6-31G(d,p) method (15.37–16.02 = −0.65 kcal/mol) was verified with the B3LYP/6-311++G(d,p) methods to be 1.22 kcal/mol in favour of TGE. This result ensures us that TGE is a better antioxidant than GE.

Moreover, the genistein substituted with the thiol residues at the B ring at O-4′ as well as in the A ring at O-5 seems to be a slightly better antioxidant than the genistein substituted at the A ring at O-7 (TGE). This is a promising line for further studies on new antioxidants.

The predictive activity of new derivatives of naturally available compounds based on structural changes is important for considering such molecules as drug candidates and for creating new structures with promising properties. Flavonoids are well-known antioxidants, and their redox properties can be related to their pharmacological activity. The current state of knowledge on the biological activity of flavonoid compounds indicates unequivocally that their positive effect on the human body results mainly from antioxidant properties. The antioxidant activity of individual flavonoids depends on the number of hydroxyl groups and their position. Para and ortho positions enhance these properties. However, observations in in vitro studies are not always confirmed in in vivo studies, particularly with regard to the number of -OH groups [50,51]. The antioxidant activity of flavonoids is possible through different mechanisms of action, among others; also, indirectly, flavonoids can chelate transition metal ions (copper, iron), which prevents the formation of reactive hydroxyl radicals in cells. The presence of “soft” donor atoms, such as nitrogen or sulfur (thiogenistein), can determine the formation of active complexes with iron ions, which are characterized by a strong anticancer activity. In contrast, compounds contained in their structure, “hard” donor atoms such as oxygen, lead to the formation of redox-inactive complexes by blocking the coordination sphere [52,53].

## 3. Materials and Methods

### 3.1. Materials

The reagents used in the study were obtained from POCh (Gliwice, Poland) and Sigma-Aldrich (Saint Louis, MO, USA). They were of the highest purity and used without prior purification. All solutions were prepared with deoxidized water, distilled, and cleaned in a “Milli-Q” filter apparatus (Millipore Corporation, Bedford, MA, USA). Its final resistance was 18.2 MΩ/cm.

TGE was synthesized in Łukasiewicz Research Network–Industrial Chemistry Institute (Łukasiewicz-ICHP), Warsaw, Poland. Ammonium formate buffer was obtained from Sigma-Aldrich (99%, LC–MS grade, Saint Louis, MO, USA). The pH of the solutions used in the experiments was determined using a commercially available Mettler Toledo (Greifensee, Switzerland) pH meter.

The monolayers of the TGE compound on the gold electrodes were prepared in the self-assembly process. First, the monolayers of the TGE compound were prepared on a gold surface by immersing the purified gold electrodes in ethanolic solutions containing 1 mM TGE. After their removal, the electrodes were rinsed thoroughly with ethanol, water, and ethanol to wash off the physically adsorbed molecules and left to dry in the air.

### 3.2. Antioxidant Study

#### 3.2.1. ABTS Radical Scavenging Assay

The ABTS radical scavenging activity was measured based on the following slightly modified method proposed by Re and co-workers [54]. Briefly, the ABTS-mixture solution was prepared by mixing 2.45 mM potassium peroxodisulfate (the final concentration) and 7 mM ABTS water solutions. For the formation of free radicals ABTS^·+^, the final solution was left in the dark at room temperature for 16 h. The obtained radical was stable in this form for about 72 h and stored in the dark at room temperature. For the study of thiogenistein scavenging activity, ABTS^·+^ solution was diluted with ethanol to an absorbance at 734 nm reached 1.3 (±0.03). Stock solutions of genistein and thiogenistein in DMSO were diluted such that, after the introduction of each dilution into the 1 mL of ABTS^·+^ solution, they reached a final concentration of 100 μM, 50 μM, 25 μM, and 10 μM (<1% DMSO). The absorbance of the resulting solutions was measured at 734 nm in a microplate reader POLARstar Omega (BMG Labtech, Ortenberg, Germany) after 5, 10, 15, 30, and 45 min after dark incubation. Genistein was used as a reference standard. The percent sweep (RSA %) was calculated according to the equation below:

$$\mathrm{RSA}\%=\frac{\left({\mathrm{ABTS}}^{•+}\mathrm{absorbance}-\mathrm{sample}\;\mathrm{absorbance}\right)\cdot 100}{{\mathrm{ABTS}}^{•+}\mathrm{absorbance}}\left[\%\right]$$

The results were expressed as mean ± standard deviation (SD) from 3 samples for each experimental group.

#### 3.2.2. H_2_DCF-DA ROS Detection Assay

The level of generated ROS was measured in the murine macrophage cells line (RAW 264.7, ATTC). Cells were grown in Dulbecco’s Modified Eagle’s Medium (DMEM, Sigma), with the addition of 10% (*v/v*) fetal bovine serum (FBS, Sigma). Cells were seeded at 6 × 10^4^ cells per well in a 96-well plate and incubated for 24 h in a humidified atmosphere at 37 °C with 5% CO_2_. Then, cells were treated with different concentrations of GE and TGE and with 10 μg/mL of LPS. Oxidative species produced by cells incubated with GE and TGE for 24 h were detected with the dichloro-dihydrofluorescein diacetate (DCFH-DA) assay. The DCFH-DA is internalized within the cells in reduced form. In the presence of ROS, the probe is oxidized into the fluorescent form, thus, the measured intensity is proportional to the intracellular oxidative stress. The RSA%, and ROS generation levels were evaluated following the manufacturer’s protocols for absorbance, fluorescence, or luminescence measurement using a microplate reader POLARstar Omega (BMG Labtech, Ortenberg, Germany). The results were expressed as mean ± standard deviation (SD) from 6 samples for each experimental group.

### 3.3. In Vitro Study–Cell Viability, Cytotoxicity

Two models of neoplastic cells, breast (MCF-7 estrogen positive and MDA-MB-231 estrogen negative cells) were selected for the study. Breast cancer cells, MCF-7 (ATTC), were grown in the Eagle’s Minimum Essential Medium (EMEM) with the addition of 10% (v/v) fetal bovine serum (FBS, ATTC) and 0.1 mg/mL human recombinant insulin (Gibco). MDA-MB-231 (ATTC) cells were maintained of complete growth medium Leibovitz’s L-15 supplemented by 10% FBS (ATTC).

Genistein and thiogenistein in a DMSO stock solution were each diluted in the growth culture medium and added in quintuplicates to the wells in the final concentration (100, 50, 25, 12.5, and 6.25 µM). The maximum content of DMSO was <1%. Both cell lines were incubated with the addition of drugs for 6 h, 24 h, and 72 h after that, the cells were washed three times in Hank’s Balanced Salt Solution (Gibco) and analysed by the viability (PrestoBlue™, Thermo Fisher, USA) and cytotoxicity (ToxiLight, and ToxiLight 100% Lysis Control, Lonza, USA) assays. The obtained results were compared statistically using the t-test.

The cell viability, toxicity, RSA%, and ROS generation level were evaluated following the manufacturer’s protocols for absorbance, fluorescence, or luminescence measurement using a microplate reader POLARstar Omega (BMG Labtech, Ortenberg, Germany).

### 3.4. Electrochemical Measurements

TGE was injected to electrochemical treatment in 20 mM ammonium formate–acetonitrile (1:1) adjusted to pH 7.4 with ammonia solution using 1 mL syringe (Hamilton, Reno, NV, USA). The final concentration of TGE was 4 µg/mL.

Electrochemical degradation was carried out using the ROXY potentiostat (Antec Scientific, Zoeterwoude, the Netherlands) with a flow rate of 10 µL/min at room temperature and with the use of a BDD electrode in a potential range from 0 to 2500 mV. The system was composed of a palladium (Pd) counter electrode and a HyREF (Pd/H_2_) reference electrode.

### 3.5. MS Spectrometry

The MS analysis was performed on an MS/MS mass spectrometer model 4000 Q TRAP (Applied Biosystems, Concord, Ontario, Canada). The quadrupole/linear ion trap is a hybrid system in which the final quadrupole can operate as a conventional mass filter or as a linear IT (ion trap) with an axial ion inject. The analysis was performed in the EMS and EPI (Enhanced Product Ion Scan) modes with an electrospray ionization source (ESI). The EPI was performed by fragmenting the pseudomolecular ions of TGE and its degradation compounds. Spray voltage was used in a negative ionization mode at −4500 V, curtain gas pressure was set at 20 psi, the capillary temperature was set at 200 °C, and declustering potential was set at −80 V. The collision energies applied were optimized for each compound individually.

### 3.6. IR Measurements

The infrared spectra were recorded on the Nicolet iS10 FT-IR spectrometer (Thermo Scientific, Waltham, MA, USA) using ATR sampling module, on the diamond crystal, in the range from 4000 to 650 cm^−1^, with the spectral resolution of 4 cm^−1^. For one spectrum 1000 scans were recorded. All data were analyzed using Omnic software. TGE monolayers were treated with hydrogen peroxide by immersing the electrodes in a solution of concentrations of 1 mM (5 min, 15 h) and 2 mM (20 min). After this, the electrodes were removed washed with ethanol and dried.

### 3.7. Quantum Mechanical Modeling

Quantum mechanical modeling was performed using the density functional B3LYP method with medium-size Gaussian basis sets, i.e., 6-31G(d), 6-31G(d,p), and 6-311++G(d,p) for the H, C, N, O, and S atoms depending on the system investigated. Molecular geometry was optimized with the Berny algorithm implemented in the Gaussian G16 program [55]. The minimum was confirmed with all positive harmonic frequencies. All the calculations were performed on the HPC cluster in the Interdisciplinary Centre for Mathematical and Computational Modelling at the University of Warsaw, Poland.

## 4. Conclusions

In the presented study, we identified the main oxidation products of TGE generated under different environmental conditions, which can in effect determine the input of a particular structural feature to the activity. Many experimental approaches were used for the characterization of TGE after oxidation. They were based on electrochemistry and mass spectrometry (ESI-MS). The electroactive centers of TGE were identified and its oxidation mechanisms were suggested. The main reaction products formed by the oxidation of TGE were identified. These product structures indicate reactivity of the thiogenistein C, A, and B rings in reactions with peroxyl radicals and with a potential. Three types of oxidations were observed: the oxidation by oxygen attachment, the oxidation by water attachment, and dehydrogenation as the mildest form of oxidation occurring during potentiation. Four main oxidation pathways were also observed during the potential oxidation. When hydrogen peroxide was used, the oxidation mechanism is bidirectional. When both oxidizing agents were used simultaneously, two new pathways were observed in addition. It was observed that where the oxidation occurs without potential (hydrogen peroxide, potential + hydrogen peroxide) the water molecule joins the double bond of the C ring first. Where oxidation with potential was used, more water molecules were observed to attach to the rings, including the A ring. It was observed that the A ring was more favored for oxidation due to the presence of an inductive effect. To summarize, our results show that the A ring is the critical active site in TGE that contributes to its *OH trapping efficacy, which is similar to the GE MGO trapping efficacy [56].

IR-ATR studies indicated that in the case of a tightly packed monolayer, the B ring was the most susceptible to oxidation, as it is the most protruding one. It should be taken into account that in the outer molecules bounding the 2D surface, the A ring may also undergo oxidation. For this reason, it is possible to obtain different TGE oxidation products in the monolayer.

The anticancer and antioxidative activity of our new thiolated analogue of genistein was evaluated based on cell culture and the ABTS^·+^ radical cation. TGE has a high cytotoxic activity towards the human breast cancer cells (MCF-7 estrogen positive and MDA-MB-231 estrogen negative) and neutralizes the LPS-induced reactive oxygen species production better than GE, even though the thio-linker blocks one of its -OH groups. This can be interpreted by our results that the oxidation of TGE, on the one hand, takes place by passing the hydrogen atom to free oxygen radicals, and on the other hand, it can occur by attaching additional -OH groups to the frame of the molecule.

In this work, antioxidant activity was calculated by density functional theory studies for a possible correlation with the structures of TGE and products of electrochemical and oxidation conversion and investigating the mechanism of reactions of the TGE with the ABTS^+^. The model system used for these studies is reasonably predictive of antioxidant chemistry in more complex biological systems.

The understanding of the biological activity of antioxidant compounds is a very important aspect of human life. We hope that understanding these procedures will help to determine key molecular fragments involved in the subsequent biochemical processes in living organisms and give information about their role in metabolic processes and pharmacological activity [57]. We plan to study whether reactions of TGE and peroxyl radicals in lipid bilayers in vitro and in biological membranes in vitro and in vivo yield products similar to those observed in this study in a homogeneous solution system.

## Figures and Tables

**Table 1:** Summary of proposed structures of main oxidation products of TGE formed after oxidation with potential.

Product Mass, Characteristic Ions *m/z*, (rel. int., % for EPI)	No. Oxidation Product/Intensity	Proposed Metabolic Reaction-Suggested Reactions
TGE [M-H]^−^ = 387 Da; DP (−80), CE (−40) 0V, *m/z* (EPI−) 387: 369(1); 343(1); 327(1); 313(1); 299(1); 295(1); 286(1); 269(100); 268(13); 255(1); 241(1); 225(1); 224(1); 211(1); 201(1); 199(1); 196(1); 181(1); 157(1) 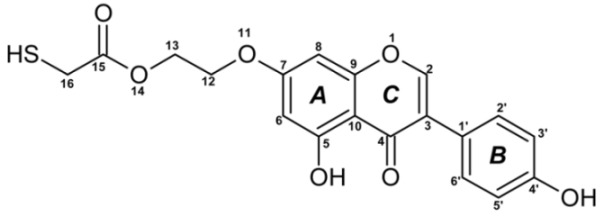 Electrode: BDD; pulse 5: pulse of 1 s/+2.5 V + pulse of 0.5 s/−0.3 V ***m/z*: EPI−**		
402: 384(4); 374(8); 368(3); 358(11); 342(3); 339(5); 329(9); 326(11); 317(7); 312(9); 303(2); 294(5); 284(20); 283(7); 268(22); 255(7); 241(4); 195(9); 177(10) M = 402 Da: 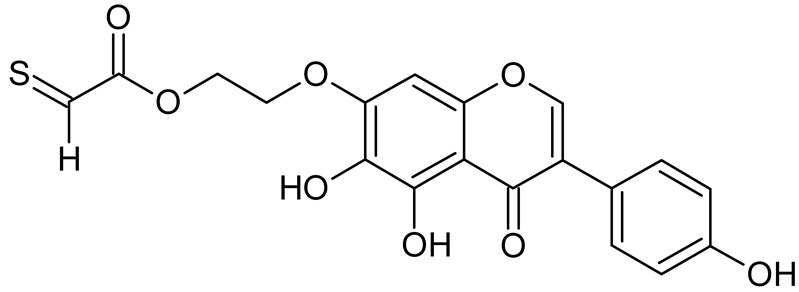 15-thioformyl-6-hydroxy-TGE (1)	1/Major	O gain (+16) + 2H loss (−2)
M = 404 Da: 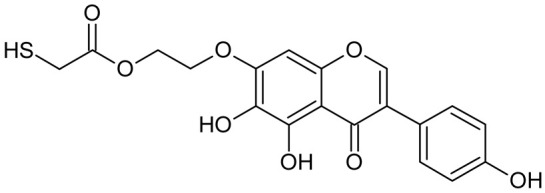 6-hydroxy-TGE (2)	2/Minor	O gain (+16)
419: 401(1); 391(57); 385(29); 375(57); 371(14); 357(14); 351 (43); 347(1); 329(43); 327(29); 315(29); 301(29); 299(29); 295 (29); 285(28); 283(42); 271(14); 269(86); 257(14); 255(28); 227(14); 195(43); 177(14); 165(57); 151(14) M = 420 Da: 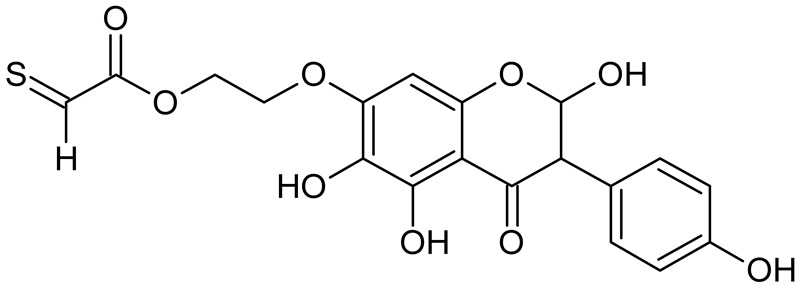 15-thioformyl-2,3-dihydro-2,6-dihydroxy-TGE (3)	3/Medium	O gain (+16) + H_2_O gain (+18) + 2H loss (−2)
M = 422 Da: 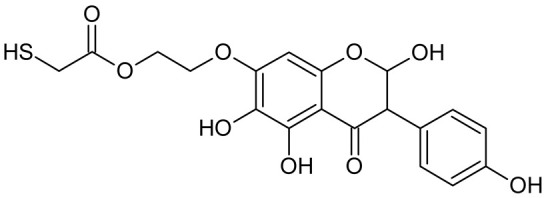 2,3-dihydro-2,6-dihydroxy-TGE (4)	4/Minor	O gain (+16) + H_2_O gain (+18)
423: 405(6); 395(25); 359(1); 339(19); 331(6); 307(1); 283 (6); 269(6); 257(100); 255(1); 229(13); 212(6); 185(13); 176(12); 120(13) M = 424 Da: 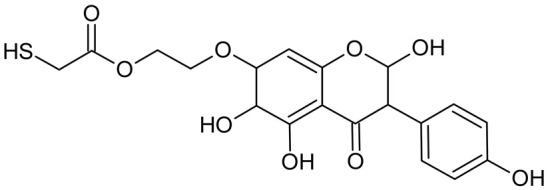 2,3,6,7-tetrahydro-2,6-dihydroxy-TGE (5)	5/Minor	2H_2_O gain (+36)
433: 415(9); 405(13); 399(4); 387(9); 369(9); 315(9); 287(9); 269(100); 243(4); 213(9); 193(17); 177(4); 139(4) M = 434 Da: 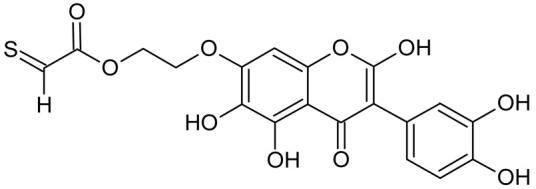 15-thioformyl-2,6,3′-trihydroxy-TGE (6) or 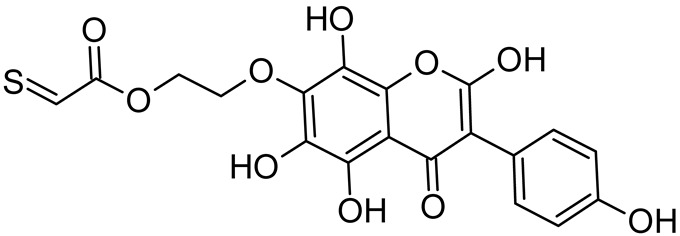	6/Medium	3O gain (+48) + 2H loss (−2)
435: 417(7); 389(7); 372(1); 363(2); 345(5); 301(10); 286(6); 280(1); 271(17); 269(100); 256 (3), 255(4); 241(2); 227(3); 212(7); 194(7); 176(1); 150(48) M = 436 Da: 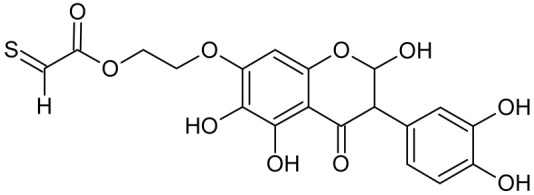 15-thioformyl-2,3-dihydro-2,6,3′-trihydroxy-TGE (7) or 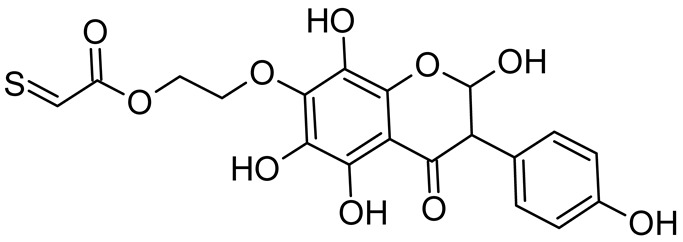 or 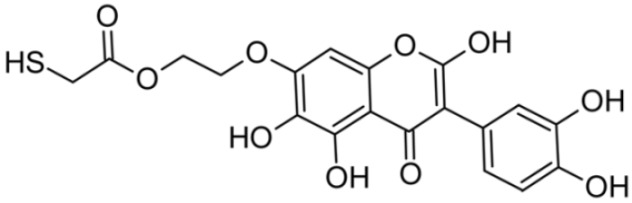 or 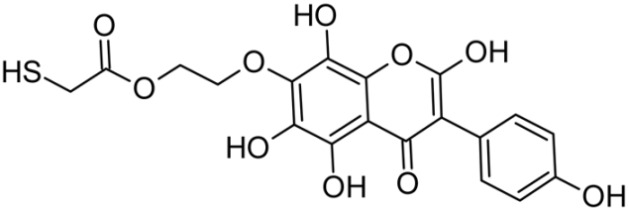	7/Medium	2H loss (−2) + 2O gain (+32) + H_2_O gain (+18) or 3O gain (+48)

**Table 2:** Summary of proposed structures of main products of TGE formed after oxidation with 0.34 M H_2_O_2_, 0 V.

Product Mass, Characteristic Ions, *m/z*, (rel. int., % for EPI)	No. Oxidation Product/Intensity	Proposed Metabolic Reaction–Suggested Reactions
TGE [M-H]^−^ = 387 Da ***m/z*: EPI−**		
435: 349(1); 286(1); 269(100); 241(1); 224(1); 213(1); 201(1); 181(1); 165(1); 157 (1); 121(2) M = 436 Da: 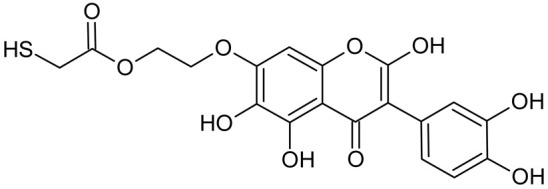 2,6,3′-trihydroxy-TGE (8)	8/Major	3O gain (+48)
M = 438 Da: 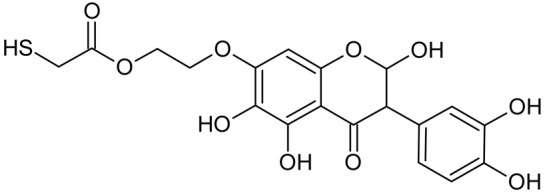 2,3-dihydro-2,6,3′-trihydroxy-TGE (9)	9/Minor	2O gain (+32) + H_2_O gain (+18)
451: 423(2); 285(100); 283(2); 269(3); 257(5); 229(2); 217(3); 213(1); 121(2) M = 452 Da: 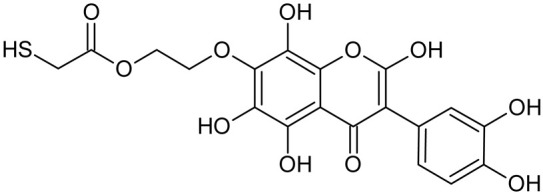 2,6,8,3′-tetrahydroxy-TGE (10)	10/Minor	4O gain (+64)
453: 438(67); 435(17); 425(50); 409(17); 407(33); 395(17); 364(100); 338(17); 309(33); 295(17); 287(67); 285(100); 269(17); 241(17); 229(17); 123(33) M = 454 Da: 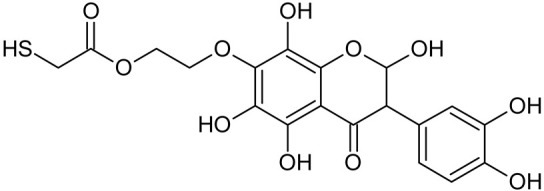 2,3-dihydro-2,6,8,3′-tetrahydroxy-TGE (11)	11/Medium	3O gain (+48) + H_2_O gain (+18)
467: 450(2); 449 (1); 434(61); 433(1); 421(2); 405(1); 370(4); 369(1); 328(13); 317(2); 301(10); 284(20); 283(17); 268(100); 267(7); 255(6); 239(4); 211(2); 193(6); 151(3); 121(3) M = 468 Da: 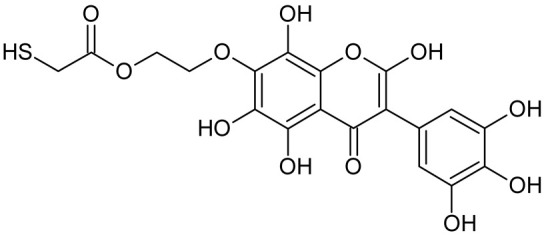 2, 6, 8,3′,5′-pentahydroxy-TGE (12)	12/Minor	5O gain (+80)
469: 454(5); 441(7); 437(30); 425(6); 423(20); 411(5); 409(100); 397(4); 383(8); 380(4); 355(2); 353(20); 339(5); 325(13); 317(3); 311(7); 307(11); 303(7); 295(2); 285(28); 283(4); 269(8); 257(3); 241(4); 225(1); 199(2); 185(2); 171(6); 151(4); 147(2); 121(2) M = 470 Da: 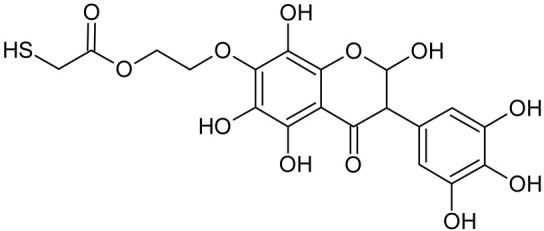 2,3-dihydro-2,6,8,3′,5′-pentahydroxy-TGE (13)	13/Medium	4O gain (+64) + H_2_O gain (+18)
485: 467(1); 451(3); 439(2); 437(30); 435(11); 409(100); 407(3); 393(6); 375(3); 350(3); 319(2); 301(5); 285(5); 269(5); 257(2); 243(1); 227(2); 199(2); 183(1); 151(2); 143(1); 121(2) M = 486 Da: 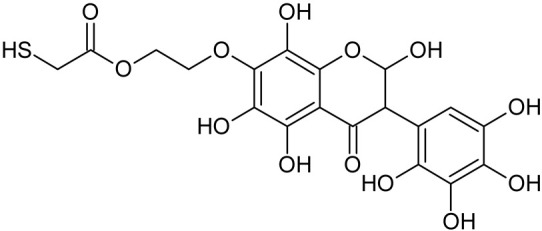 2,3-dihydro-2,6,8,3′,5′,6′-hexahydroxy-TGE (14)	14/Medium	5O gain (+80) + H_2_O gain (+18)
501: 483(1); 473(1); 453(11); 437(7); 425(10); 409(100); 407(5); 393(5); 379(5); 364(8); 352(3); 336(3); 309(3); 291(2); 282(5); 268(2); 155(1); 139(1); 123(1) M = 502 Da: 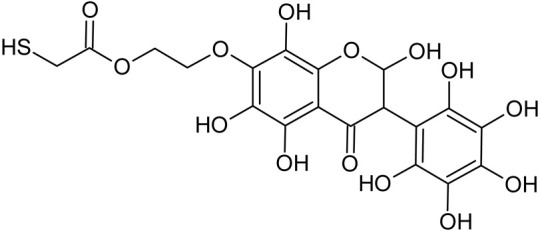 2,3-dihydro-2,6,8,2′,3′,5′,6′-heptahydroxy-TGE (15)	15/Minor	6O gain (+96) + H_2_O gain (+18)
520: 502(3); 476(6); 474(98); 457(58); 448(10); 435(83); 430(29); 429(6); 406(5); 404(8); 390(5); 372(3); 358(6); 353(6); 338(5); 326(6); 306(3); 283(4); 269(100); 197(5); 177(8); 121(10) M = 520 Da: 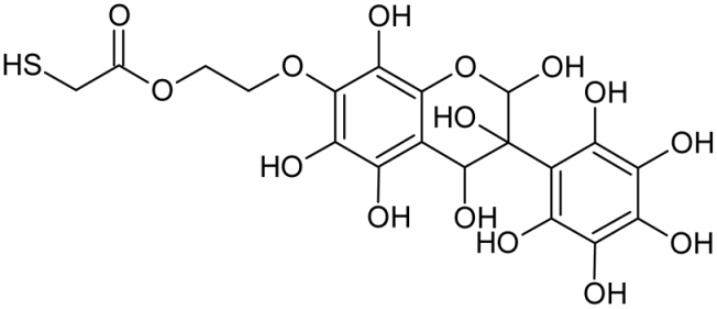 2-hydro-2,3,4,6,8,2′,3′,5′,6′-nonahydroxy-TGE (16)	16/Minor	6O gain (+96) + 2H_2_O gain (+36)
536: 518(1); 502(1); 490(3); 473(11); 455(1); 454(1); 435(86); 429(1); 402(1); 384(1); 382(1); 370(3); 353(3); 338(1); 308(1); 285(2); 269(100); 214(2); 165(1); 121(2) M = 536 Da: 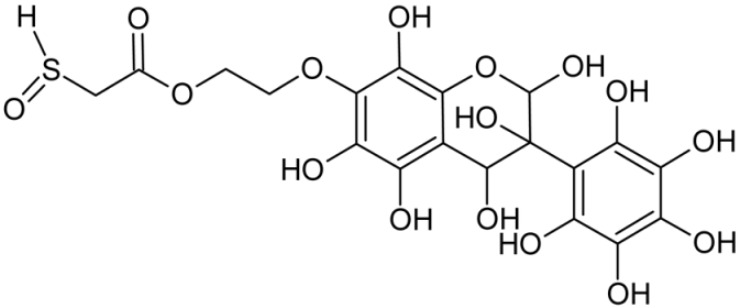 16-sulfenic-2-hydro-2,3,4,6,8,2′,3′,5′,6′-nonahydroxy-TGE (17)	17/Minor	7O gain (+112) + 2H_2_O gain (+36)

**Table 3:** Summary of proposed structures of main oxidations products of TGE formed after 0.34 M H_2_O_2_ and potential.

Product Mass, Characteristic Ions *m/z*, (rel. int., % for EPI)	No. Oxidation Product/Intensity	Proposed Metabolic Reaction-Suggested Reactions
TGE [M-H]^−^ = 387 Da ***m/z*: EPI−**		
423: 395(7); 339(7); 307(27); 287(3), 269(3); 257(100); 229(10); 213(1); 185(10); 177(3); 159(1); 143(1); 135(1); 121(10) M = 424 Da: 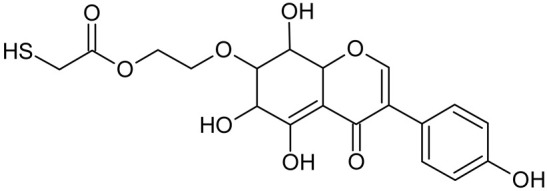 6,7,8,9-tetrahydro-6,8-dihydroxy-TGE (18)	18/Medium	2H_2_O gain (+36)
435: 349(1); 286(1); 269(100); 241(1); 224(1); 213(1); 201(1); 181(1); 165(1); 157 (1); 121(2) M = 436 Da: 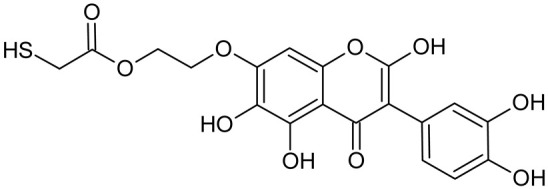 2,6,3′-trihydroxy-TGE (8)	8/Major	3O gain (+48)
M = 438 Da: 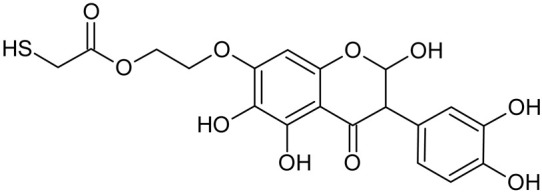 2,3-dihydro-2,6,3′-trihydroxy-TGE (9)	9/Minor	2O gain (+32) + H_2_O gain (+18)
451: 423(2); 285(100); 283(2); 269(3); 257(5); 229(2); 217(3); 213(1); 121(2) M = 452 Da: 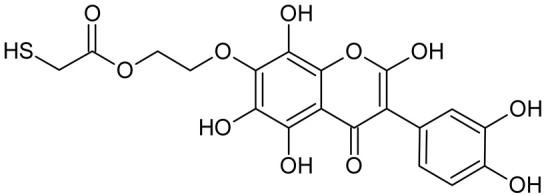 2,6,8,3′-tetrahydroxy-TGE (10)	10/Medium	4O gain (+64)
453: 438(67); 435(17); 425(50); 409(17); 407(33); 395(17); 364(100); 338(17); 309(33); 295(17); 287(67); 285(100); 269(17); 241(17); 229(17); 123(33) M = 454 Da: 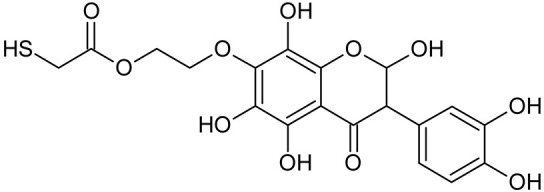 2,3-dihydro-2,6,8,3′-tetrahydroxy-TGE (11)	11/Medium	3O gain (+48) + H_2_O gain (+18)
467: 450(2); 434(61); 421(2); 370(4); 328(13); 317(2); 301(10); 284(20); 268(100); 255(6); 239(4); 211(2); 193(6); 151(3); 121(3) M = 468 Da: 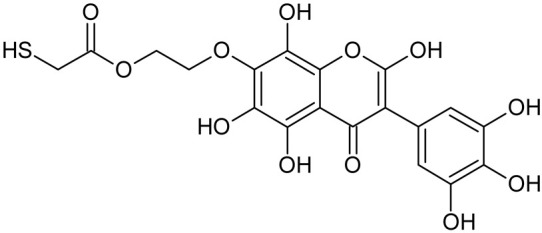 2,6,8,3′,5′-pentahydroxy-TGE (12)	12/Medium	5O gain (+80)
469: 454(5); 441(7); 437(30); 425(6); 423(20); 411(5); 409(100); 397(4); 383(8); 380(4); 355(2); 353(20); 339(5); 325(13); 317(3); 311(7); 307(11); 303(7); 295(2); 285(28); 283(4); 269(8); 257(3); 241(4); 225(1); 199(2); 185(2); 171(6); 151(4); 147(2); 121(2) M = 470 Da: 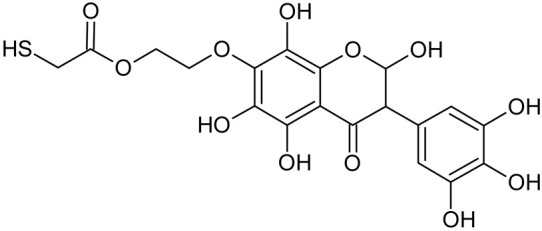 2,3-dihydro-2,6,8,3′,5′-pentahydroxy-TGE (13)	13/Minor	4O gain (+64) + H_2_O gain (+18)
473: 457(1); 409(1); 399(13); 351(13); 323(1); 313(25); 307(100); 306(12); 305(1); 295(25); 284(1); 281(13); 269(63); 239(1); 223(13); 181(1); 177(88); 165(1); 139(1); 121(50) M = 474 Da: 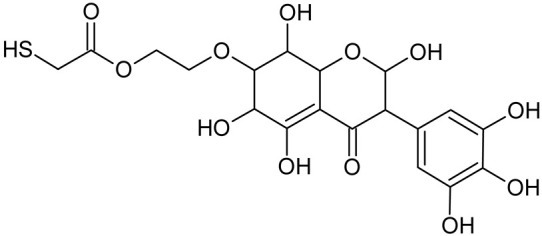 2,3,6,7,8,9-hexahydro-2,6,8,3′,5′-pentahydroxy-TGE (19)	19/Medium	2O gain (+32) + 3 H_2_O gain (+54)
485: 467(1); 451(3); 439(2); 437(30); 435(11); 409(100); 407(3); 393(6); 375(3); 350(3); 319(2); 301(5); 285(5); 269(5); 257(2); 243(1); 227(2); 199(2); 183(1); 151(2); 143(1); 121(2) M = 486 Da: 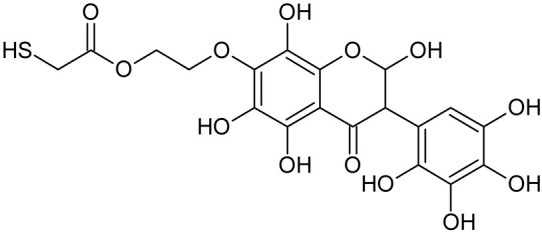 2,3-dihydro-2,6,8,3′,5′,6′-hexahydroxy-TGE (14)	14/Minor	5O gain (+80)+ H_2_O gain (+18)
501: 483(2); 473(1); 453(3); 437(9); 425(8); 409(100); 407(9); 393(5); 379(5); 364(11); 352(3); 336(1); 309(3); 291(7); 282(5); 268(1); 155(1); 139(1); 123(1) M = 502 Da: 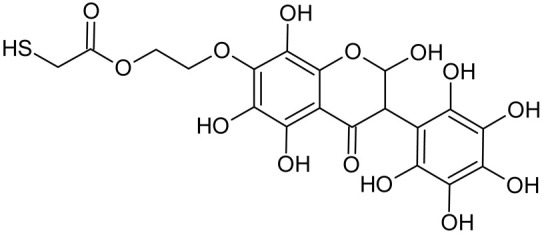 2-hydro-2,6,8,2′,3′,5′,6′-heptahydroxy-TGE (15)	15/Minor	6O gain (+96) + H_2_O gain (+18)
520: 502(6); 476(2); 474(30); 457(16); 448(7); 435(13); 430(12); 429(7); 404(5); 390(1); 358(3); 353(7); 338(4); 326(9); 306(4); 283(3); 269(100); 197(6); 177(5); 121(12) M = 520 Da: 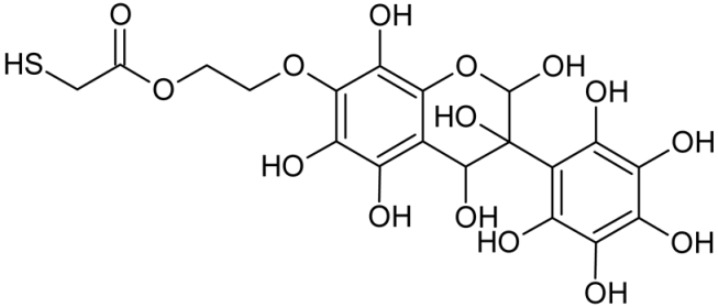 2,3-dihydro-2,3,4,6,8,2′,3′,5′,6′-nonahydroxy-TGE (16)	16/Minor	6O gain (+96) + 2H_2_O gain (+36)
536: 518(2); 502(2); 490(2); 473(4); 455(1); 435(12); 429(1); 402(1); 370(3); 353(4); 338(1); 308(1); 285(2); 269(100); 214(1); 165(1); 121(3) M = 536 Da: 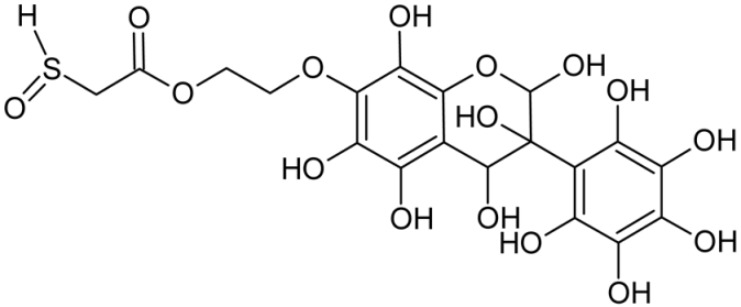 16-sulfenic-2-hydro-2,3,4,6,8,2′,3′,5′,6′-nonahydroxy-TGE (17)	17/Minor	7O gain (+112) + 2H_2_O gain (+36)

**Table 4:** The O-H bond dissociation enthalpy (BDE), in kcal/mol as predicted with the B3LYP/6-311++G(d,p) DFT method.

Compound	BDE
Phenol	82.90
Genistein	81.56
Thiogenistein	81.82
Trolox	73.91
Curcumin (*)	75.56

(*) this calculation was carried out with the B3LYP/6-31G(d,p) method.

**Table 5:** The Gibbs free energy difference for the Equation (3) reaction according to the B3LYP/6-31G(d,p) density functional calculations.

Antioxidant	ΔG, kcal/mol
Genistein (GE)	16.02
Genistein thiolated at O-7 (TGE)	15.37
Genistein thiolated at O-4′	14.88
Genistein thiolated at O-5	14.99
Genistein thiolated at O-7 by a modified thiol residue with the extra -CH_2_- group (M26P)	14.49
Trolox	11.44

## Data Availability

Not applicable.

## Supplementary Materials

The following supporting information can be downloaded at: https://www.mdpi.com/article/10.3390/ijms23147816/s1.
