# Supplementary material for: Thiogenistein—Antioxidant Chemistry, Antitumor Activity, and Structure Elucidation of New Oxidation Products
Source: Int J Mol Sci. 2022 Jul 15;23(14):7816. doi: 10.3390/ijms23147816 (PMC9315507; doi:10.3390/ijms23147816)
Supplement: Supplementary file 1 [file ijms-23-07816-s001.zip › ijms-1806819-supplementary.pdf]

Supplementary Material

Thiogenistein - Antioxidant Chemistry, Antitumor Activity, and Structure Elucidation of New Oxidation Products.

Elżbieta U. Stolarczyk[1] , Weronika Strzempek[2,3], Marta Łaszcz[1,4], Andrzej Leś[5], Elżbieta Menaszek[3], Krzysztof Stolarczyk[5,*]

1 Analytical Department, Łukasiewicz Research Network – Industrial Chemistry Institute, 8 Rydygiera Street, 01-793 Warsaw, Poland; elzbieta.stolarczyk@ichp.pl
2 Faculty of Chemistry, Jagiellonian University, 2 Gronostajowa Str., 30-387 Krakow, Poland; weronika.strzempek@doctoral.uj.edu.pl
3 Faculty of Pharmacy, Collegium Medicum, Jagiellonian University, 9 Medyczna Str., 30-068 Krakow, Poland; elzbieta.menaszek@uj.edu.pl
4 Department of Falsified Medicines and Medical Devices, National Medicines Institute, Chełmska 30/34, 00-725 Warsaw, Poland; m.laszcz@nil.gov.pl
5 Faculty of Chemistry, University of Warsaw, 1 Pasteura Street, 02-093 Warsaw, Poland; ales@chem.uw.edu.pl, kstolar@chem.uw.edu.pl
* Correspondence: kstolar@chem.uw.edu.pl; Tel. +48-22-55-26-351

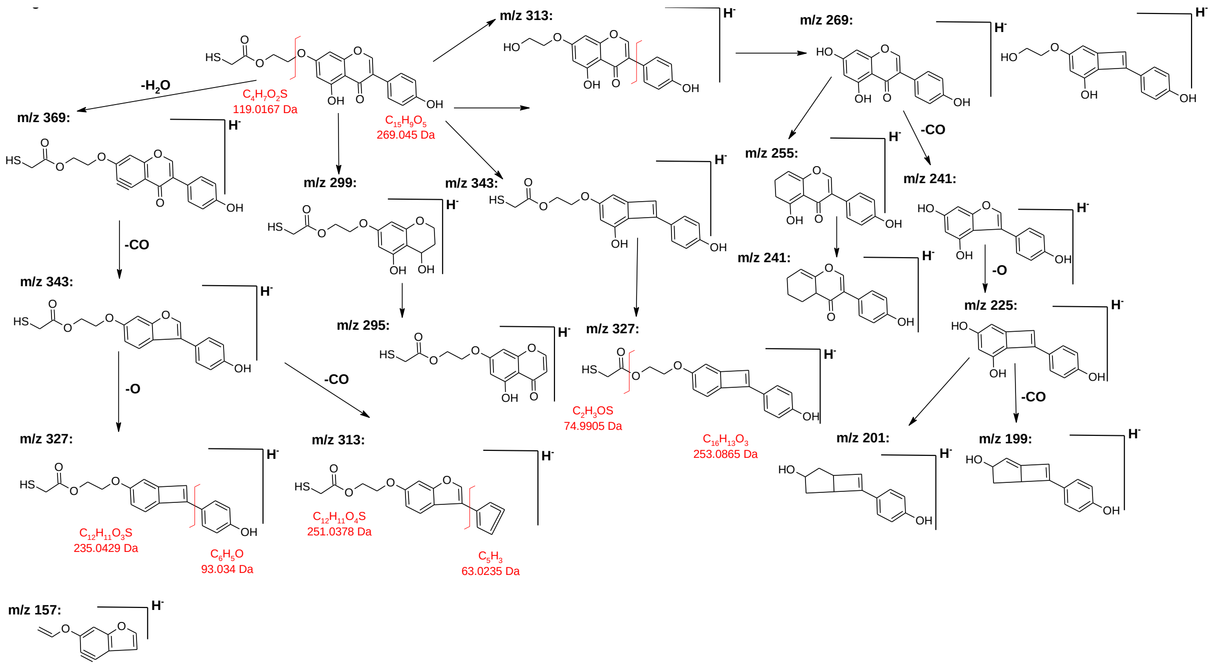

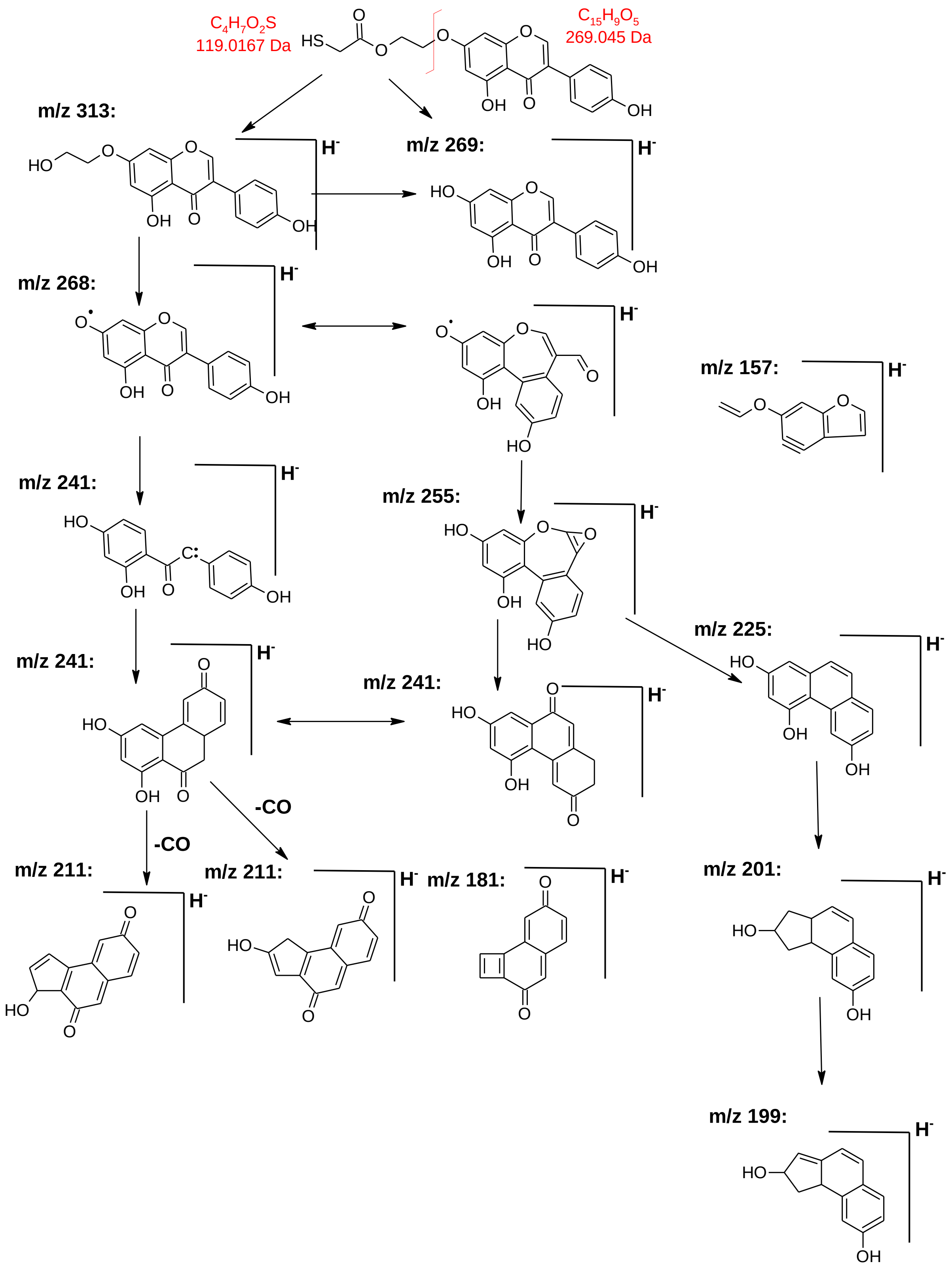

Figure S1. The proposed fragmentation pathway of TGE and structures of characteristic fragments

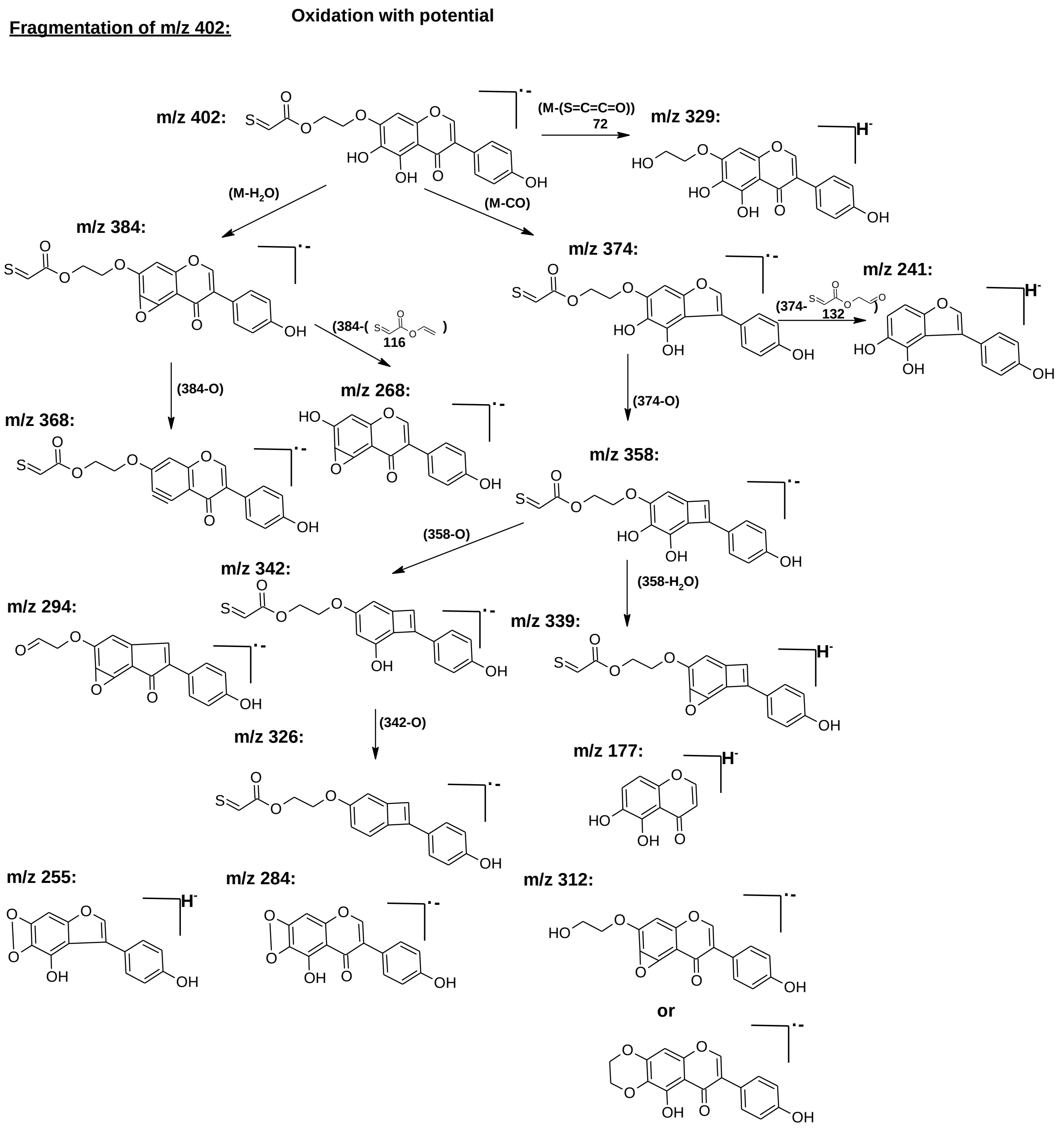

Figure S2. The proposed fragmentation pathway of compound 1 and structures of characteristic fragments.

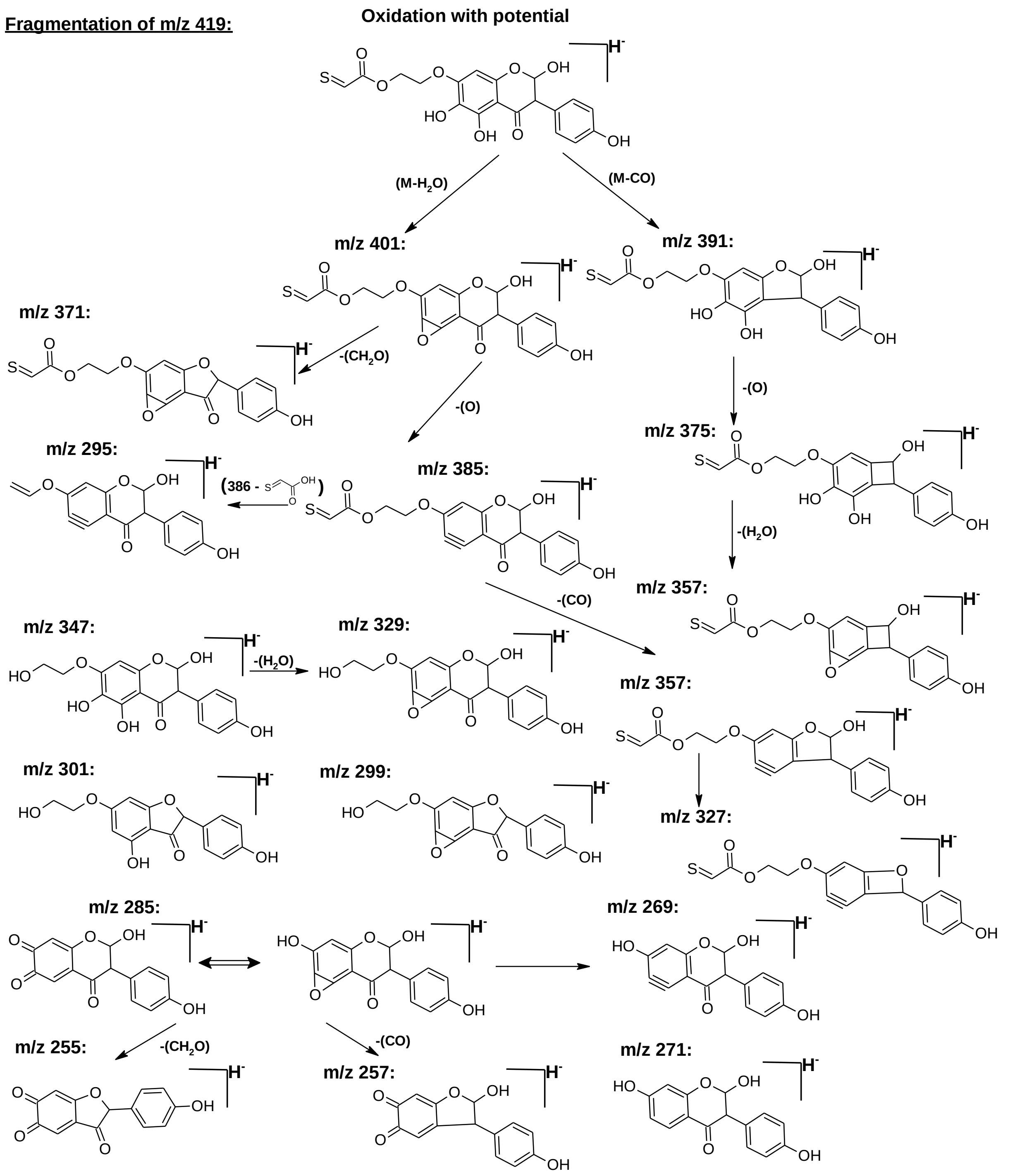

Figure S3. The proposed fragmentation pathway of compound 3 and structures of characteristic fragments.

Oxidation with potential

Fragmentation of m/z 423:

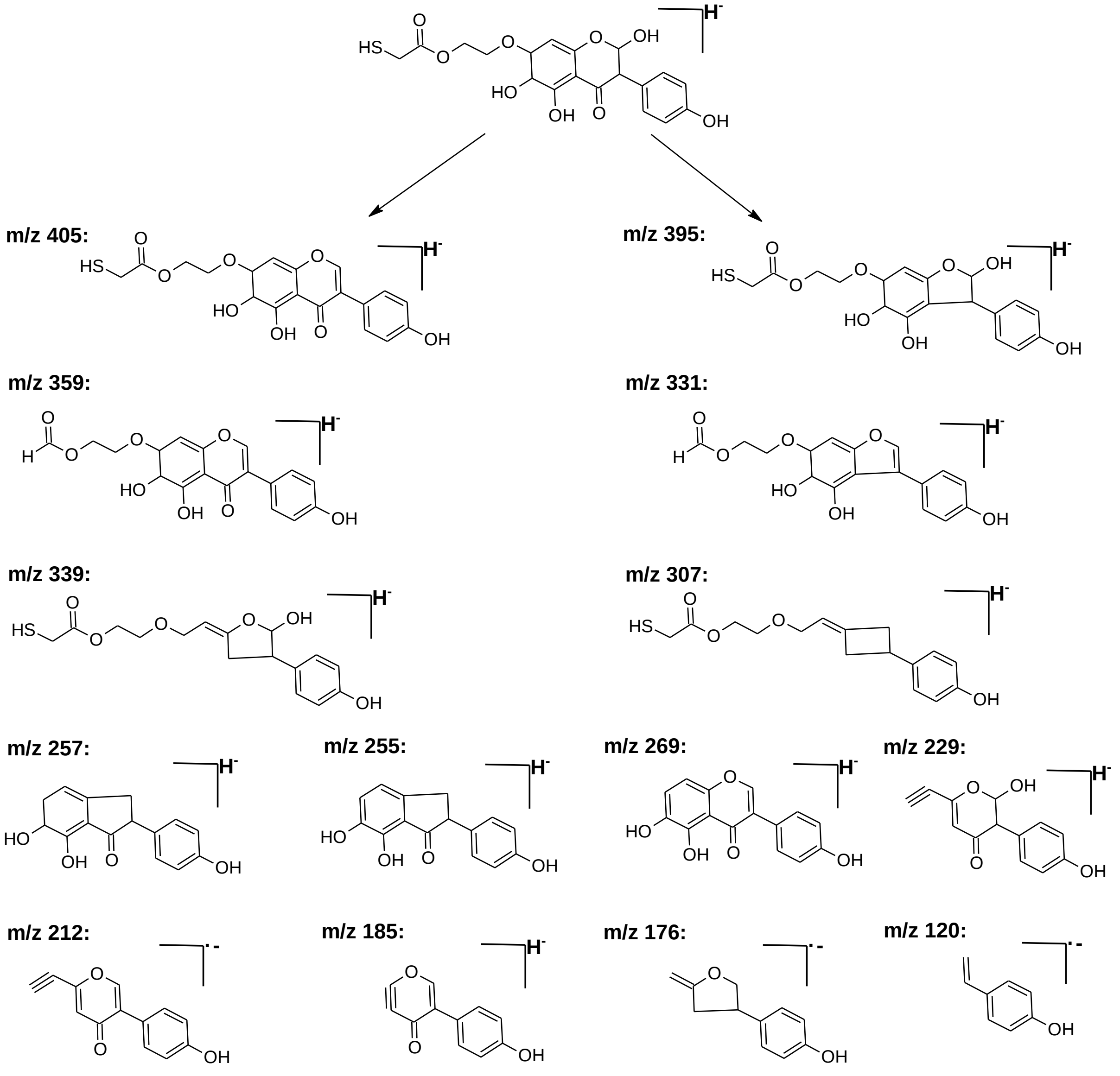

Figure S4. The proposed fragmentation pathway of compound 5 and structures of characteristic fragments

Oxidation with potential and hydrogen peroxide

Fragmentation of m/z 423:

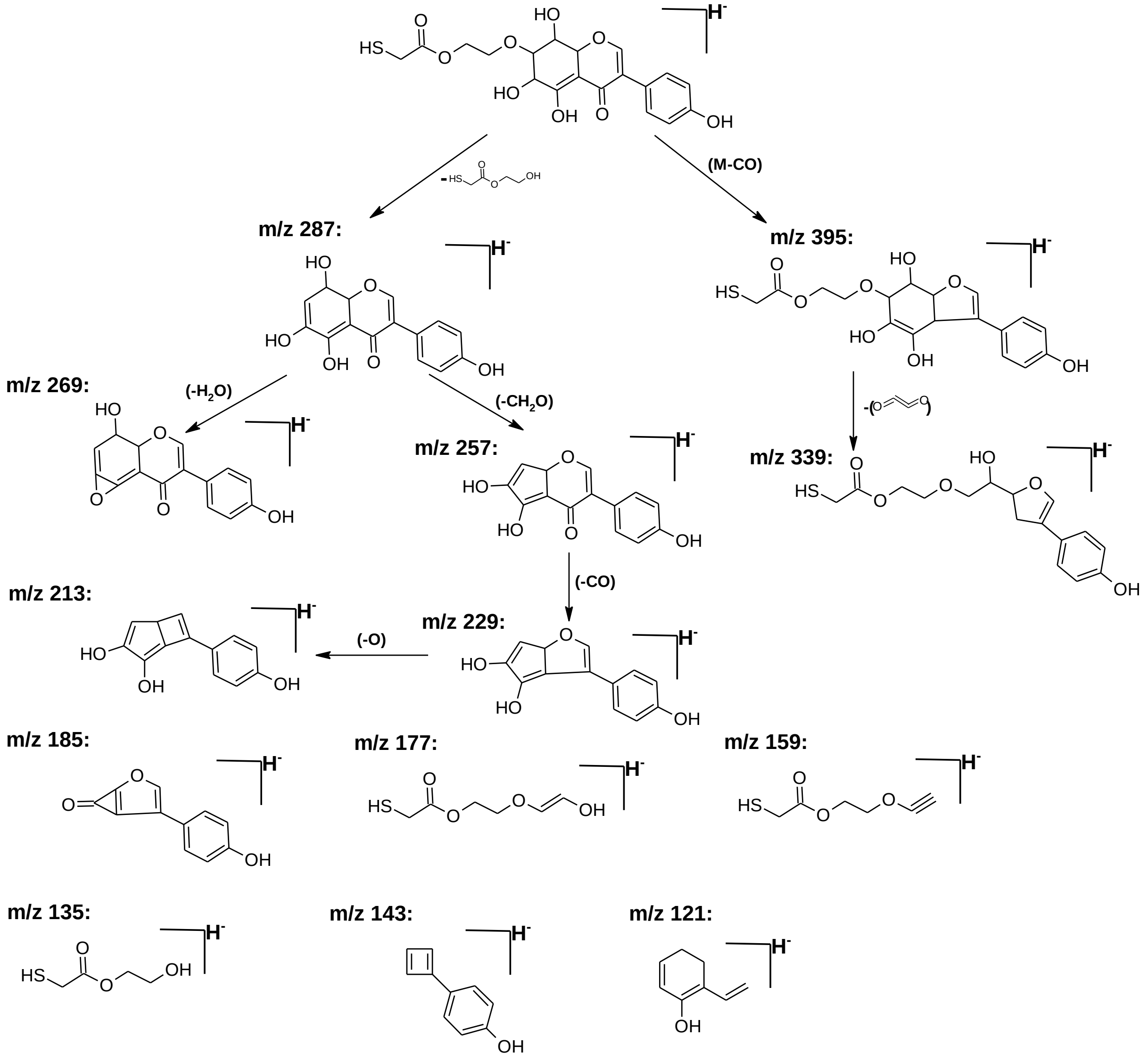

Figure S5. The proposed fragmentation pathway of compound 18 and structures of characteristic fragments

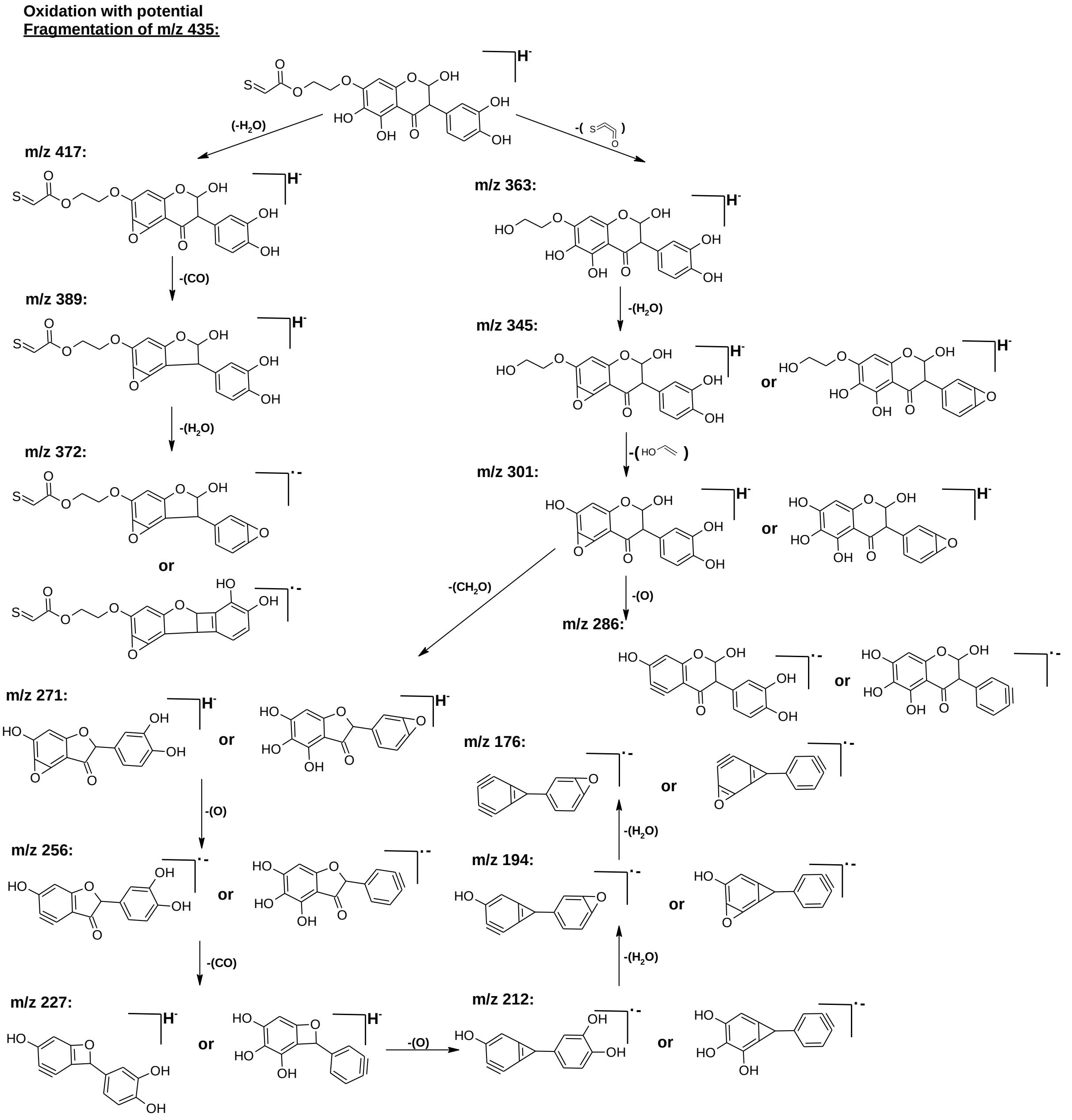

Figure S6. The proposed fragmentation pathway of compound 7 and structures of characteristic fragments.

Oxidation with potential and hydrogen peroxide

Fragmentation of m/z 435:

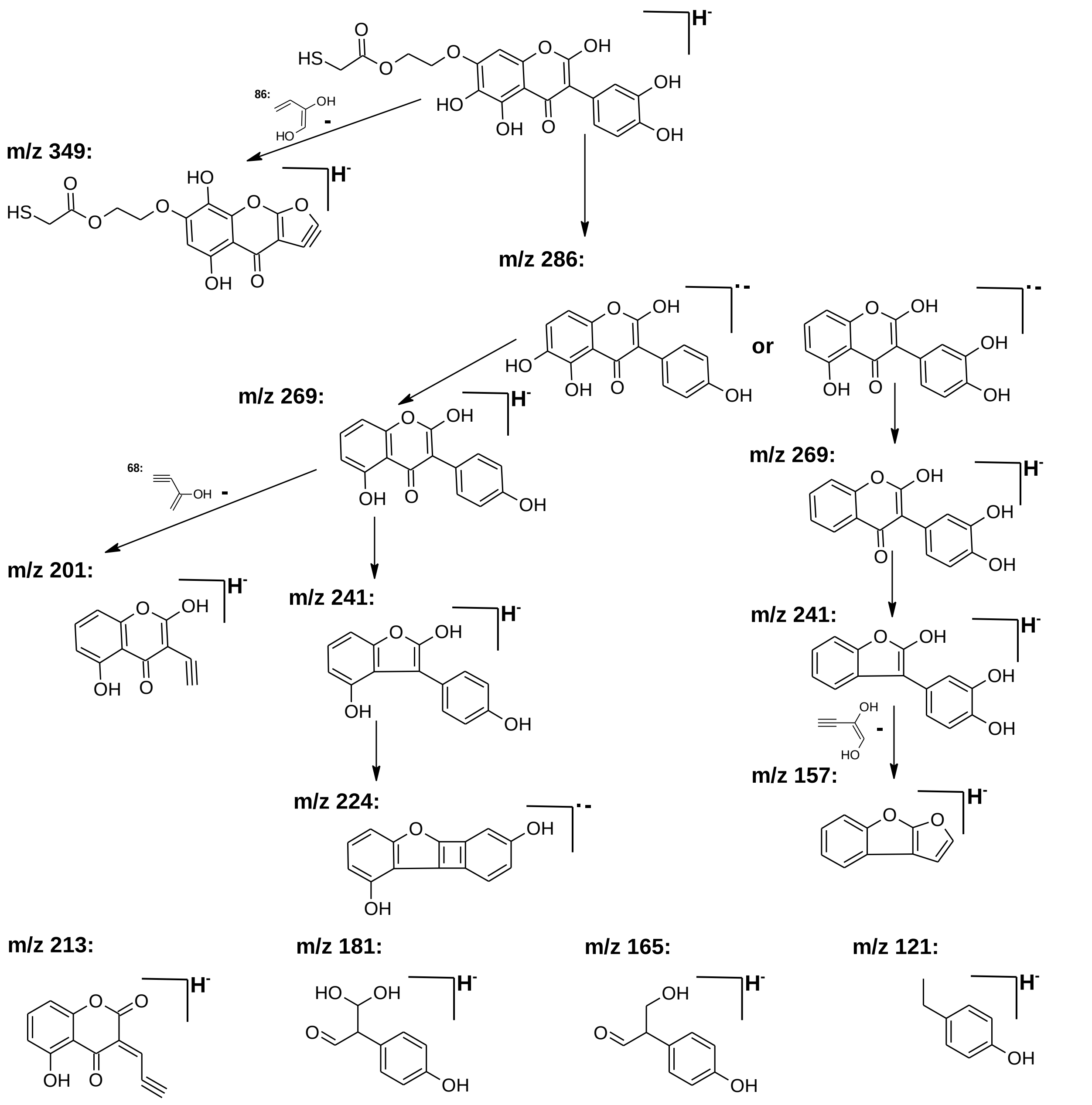

Figure S7. The proposed fragmentation pathway of compound 8 and structures of characteristic fragments.

Oxidation with hydrogen peroxide
Oxidation with potential and hydrogen peroxide

Fragmentation of m/z 451:

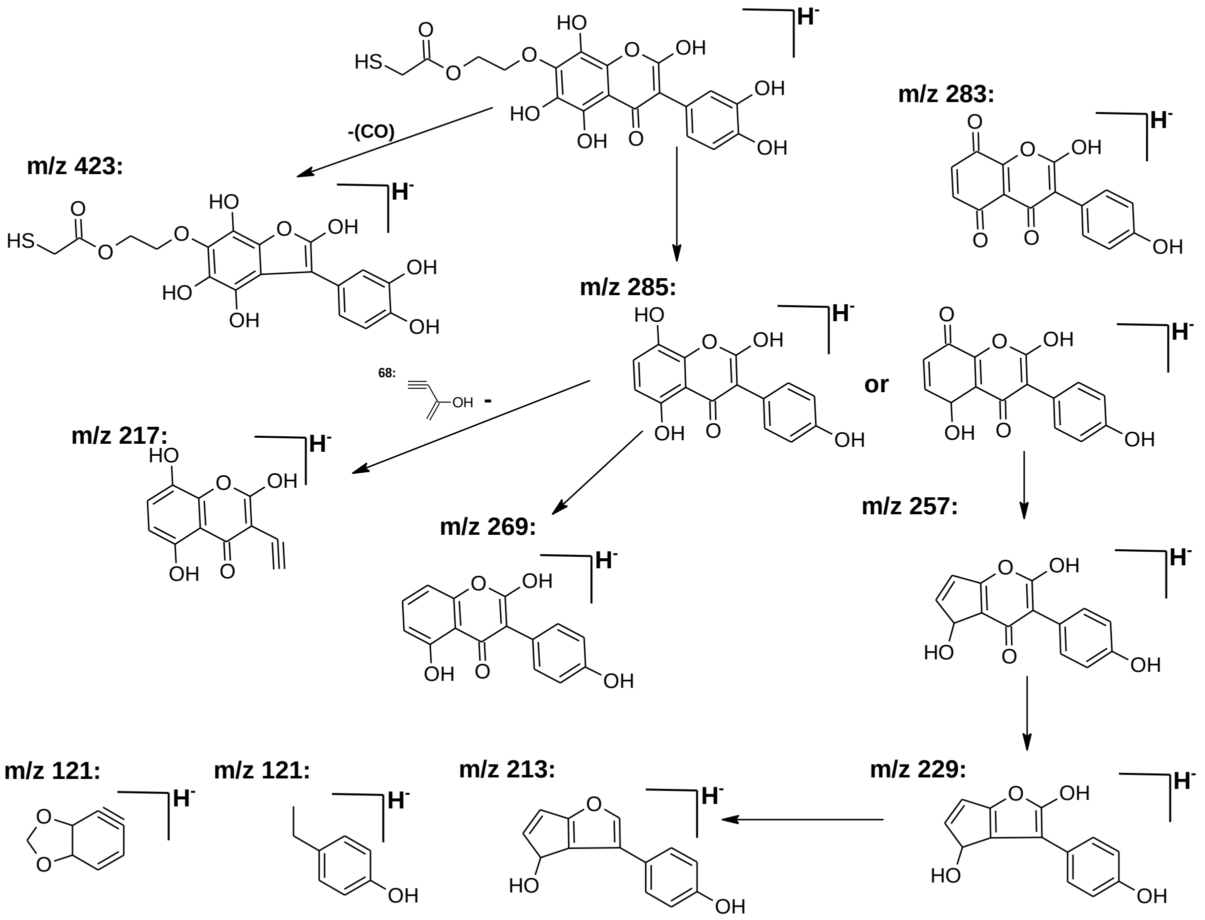

Figure S8. The proposed fragmentation pathway of compound 10 and structures of characteristic fragments

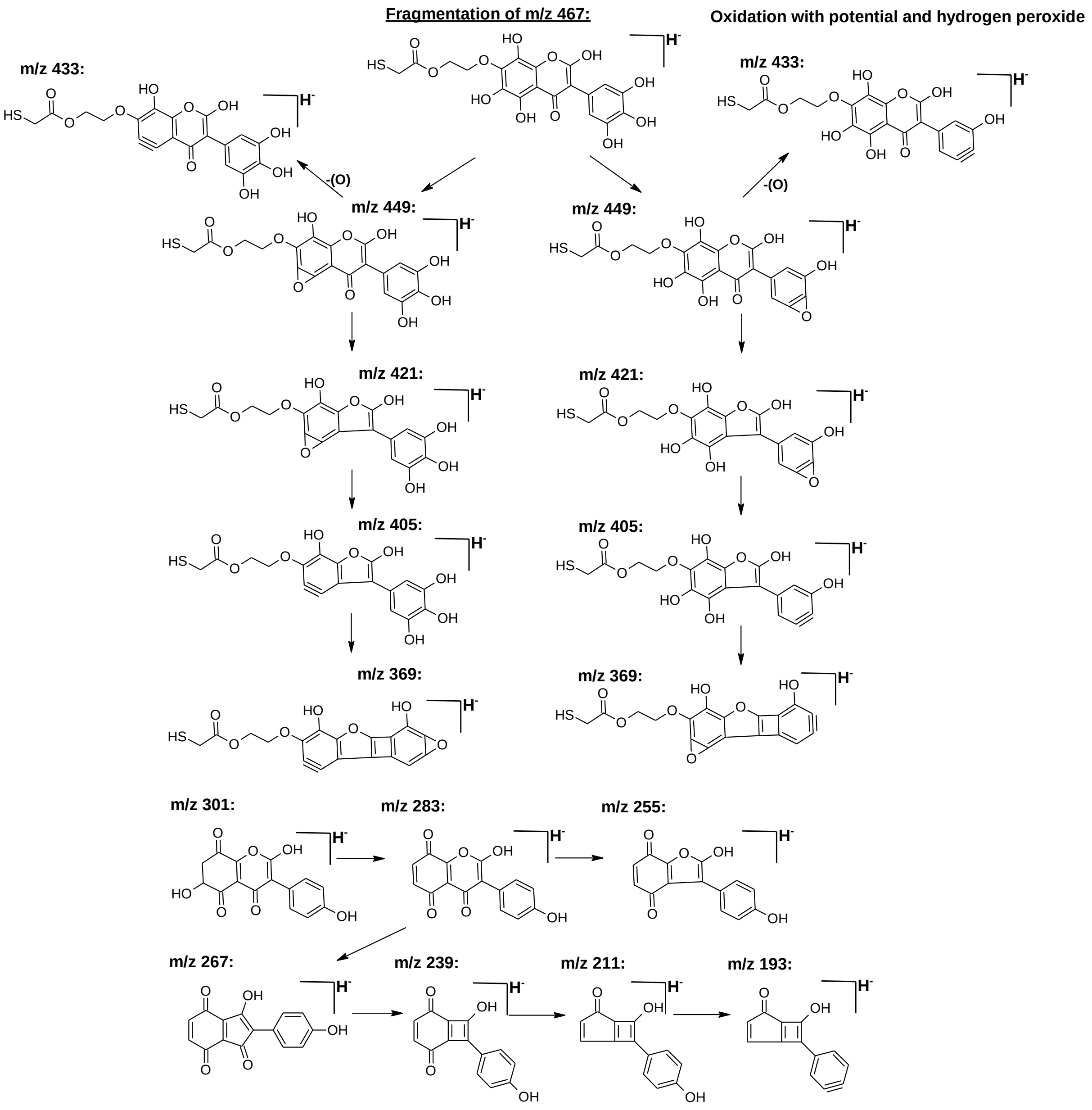

Figure S9. The proposed fragmentation pathway of compound 12 and structures of characteristic fragments

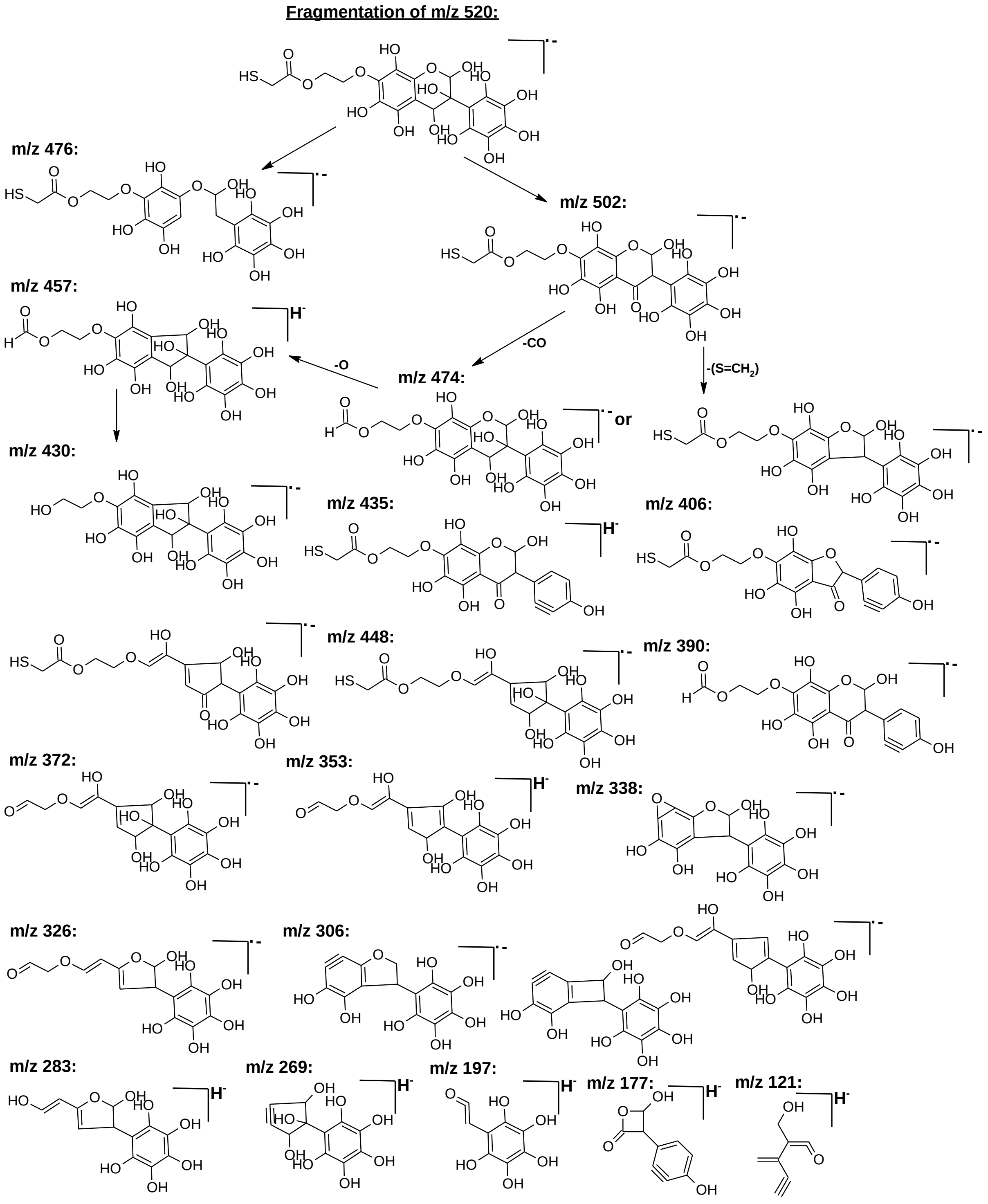

Figure S10. The proposed fragmentation pathway of compound 16 and structures of characteristic fragments

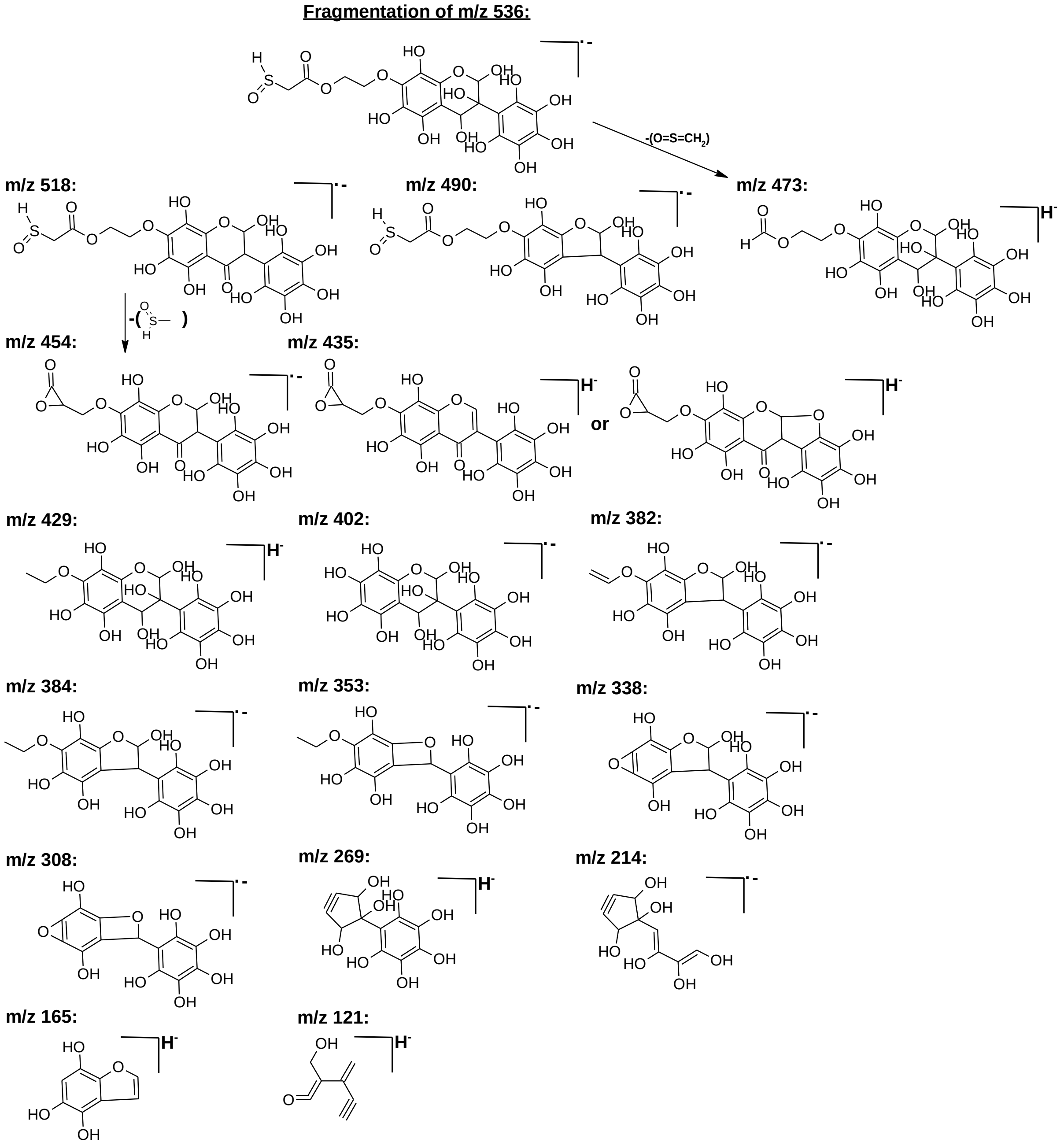

Figure S11. The proposed fragmentation pathway of compound 17 and structures of characteristic fragments

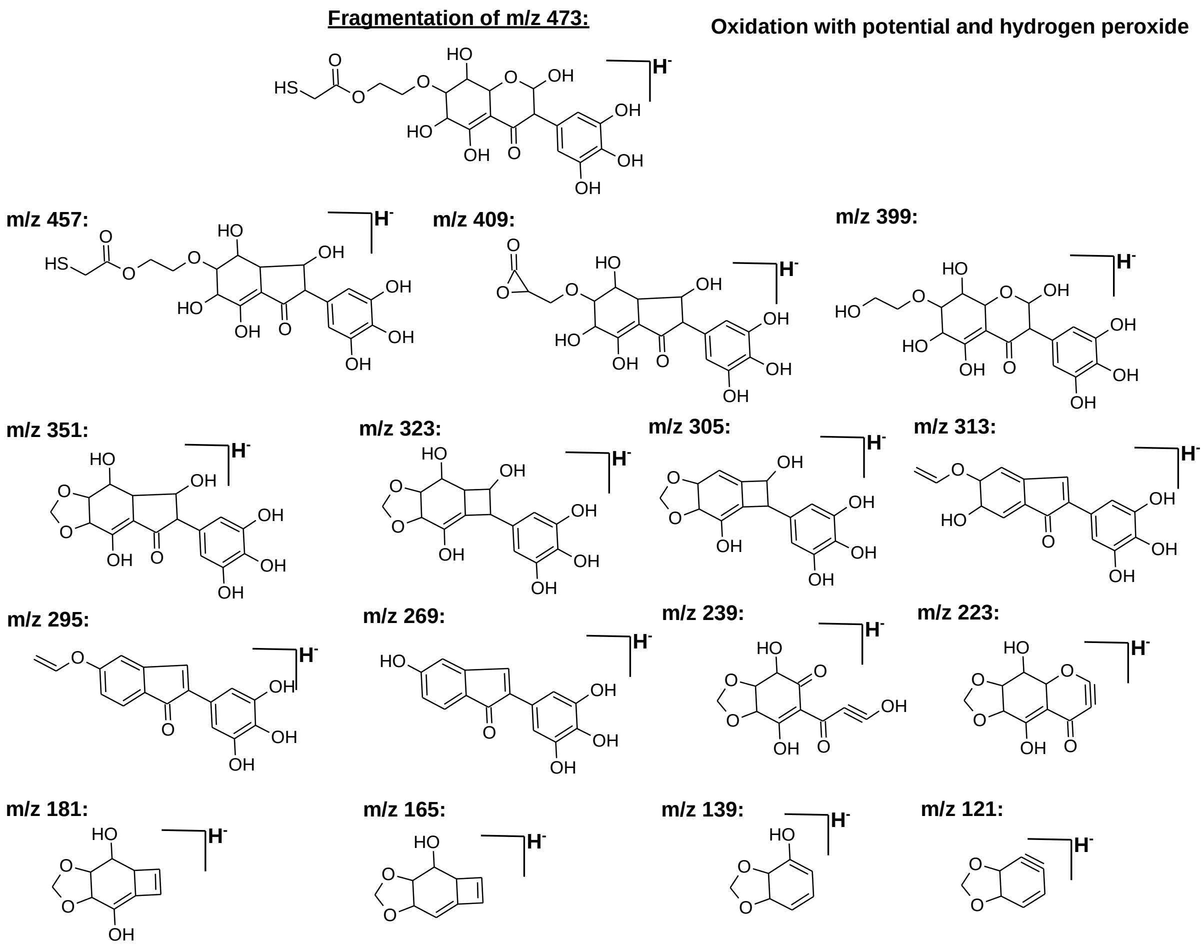

Figure S12. The proposed fragmentation pathway of compound 19 and structures of characteristic fragments

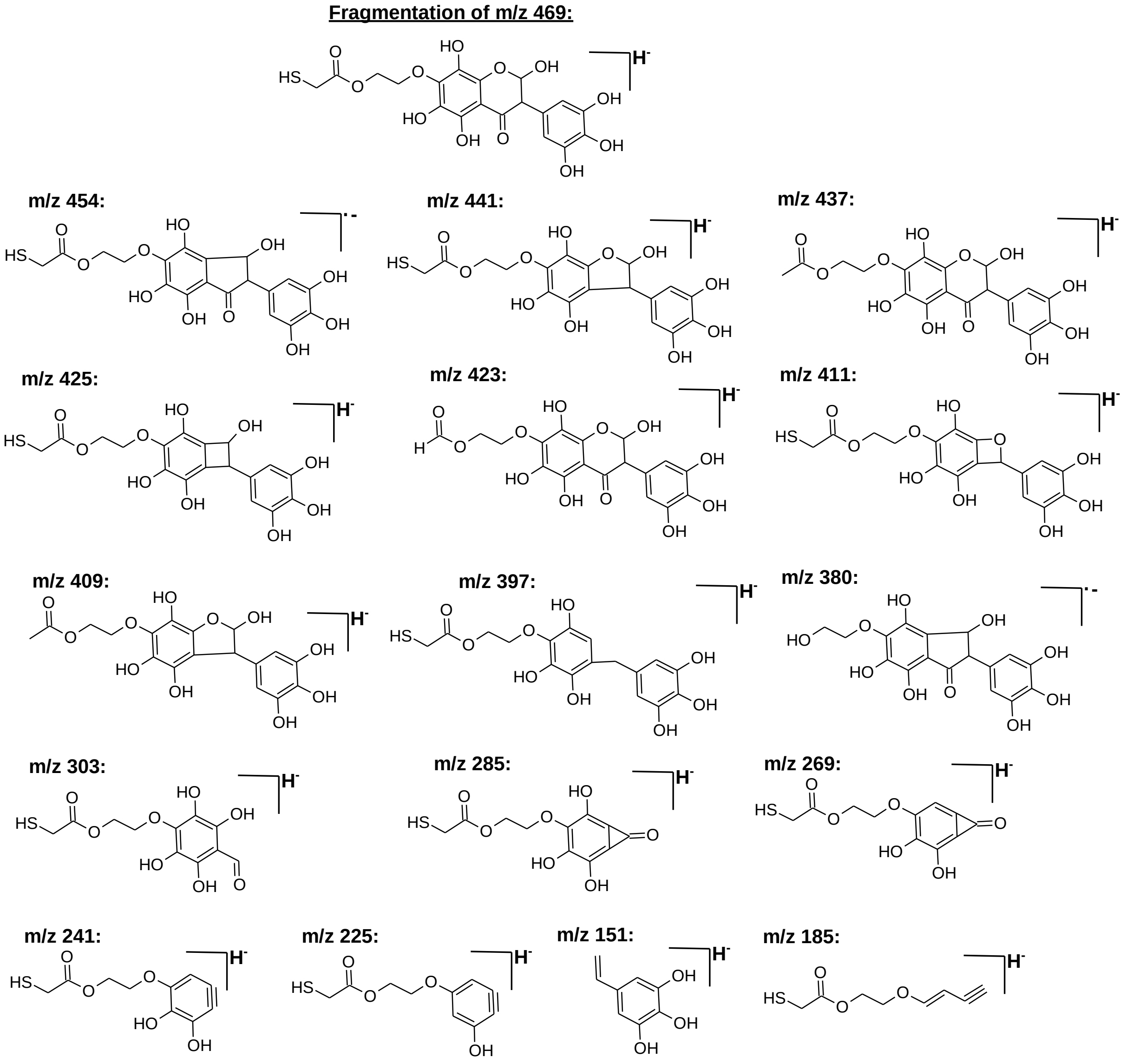

Figure S13. The proposed fragmentation pathway of compound 13 and structures of characteristic fragments

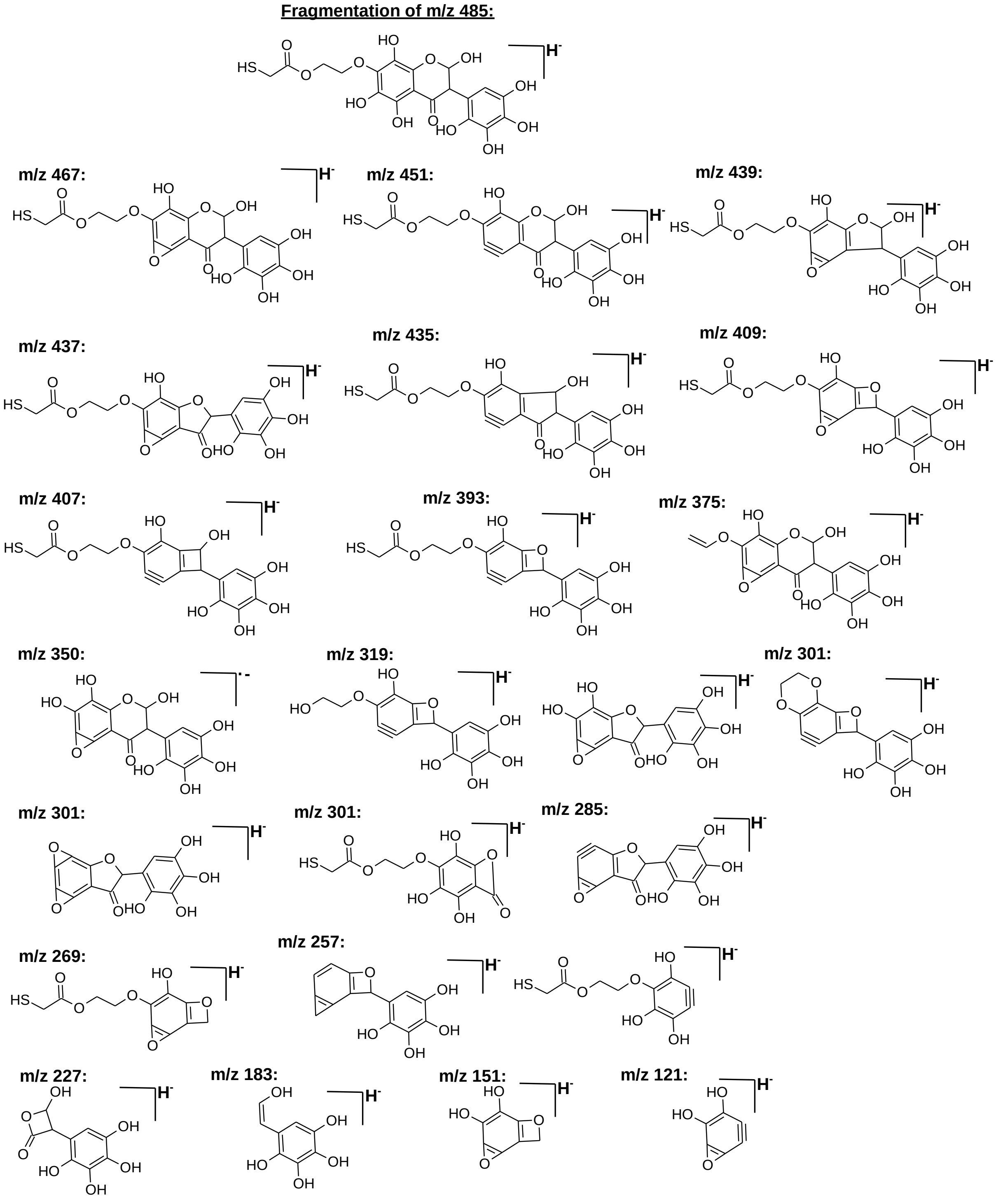

Figure S14. The proposed fragmentation pathway of compound 14 and structures of characteristic fragments

Fragmentation of m/z 501:

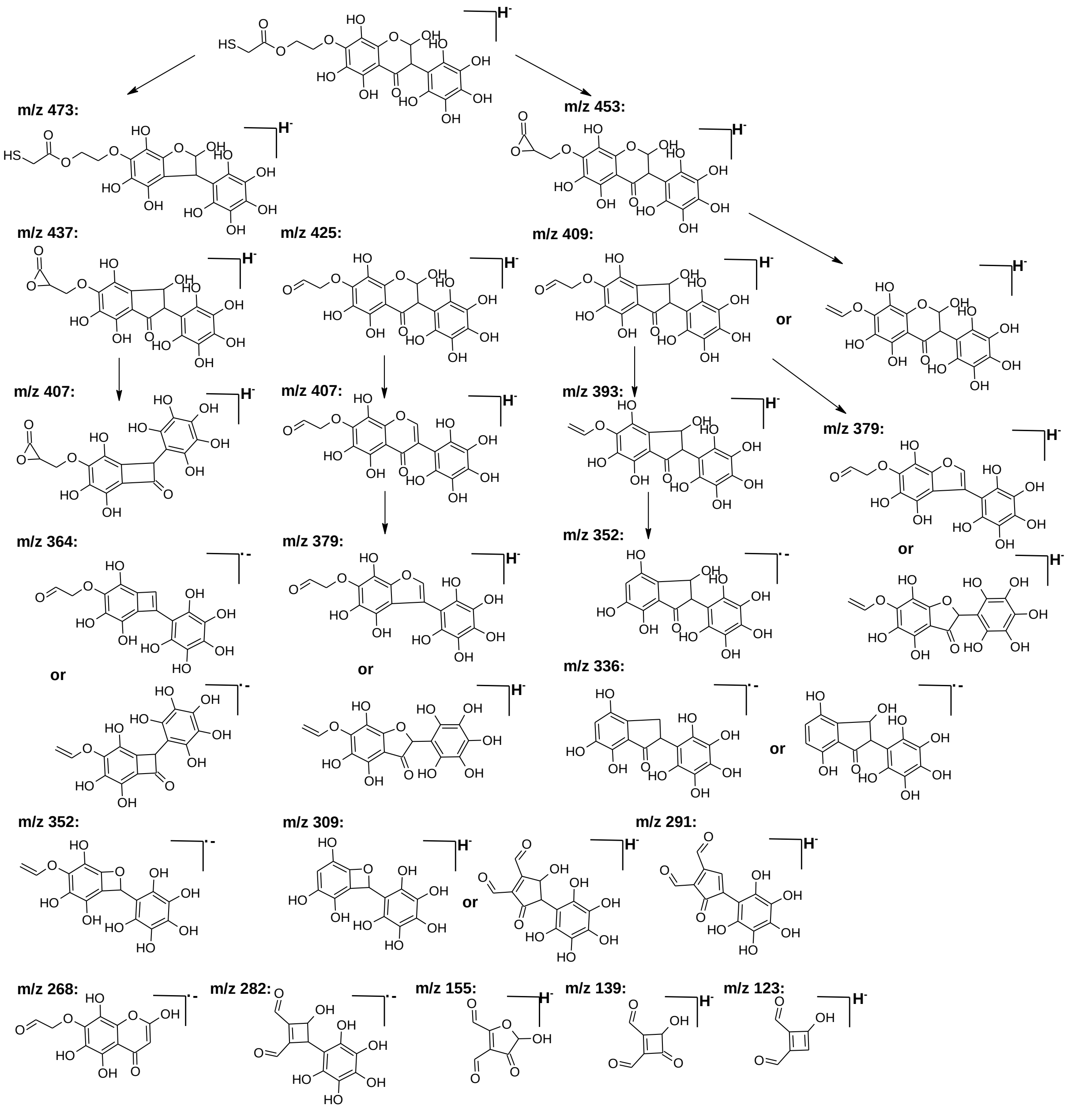

Figure S15. The proposed fragmentation pathway of compound 15 and structures of characteristic fragments

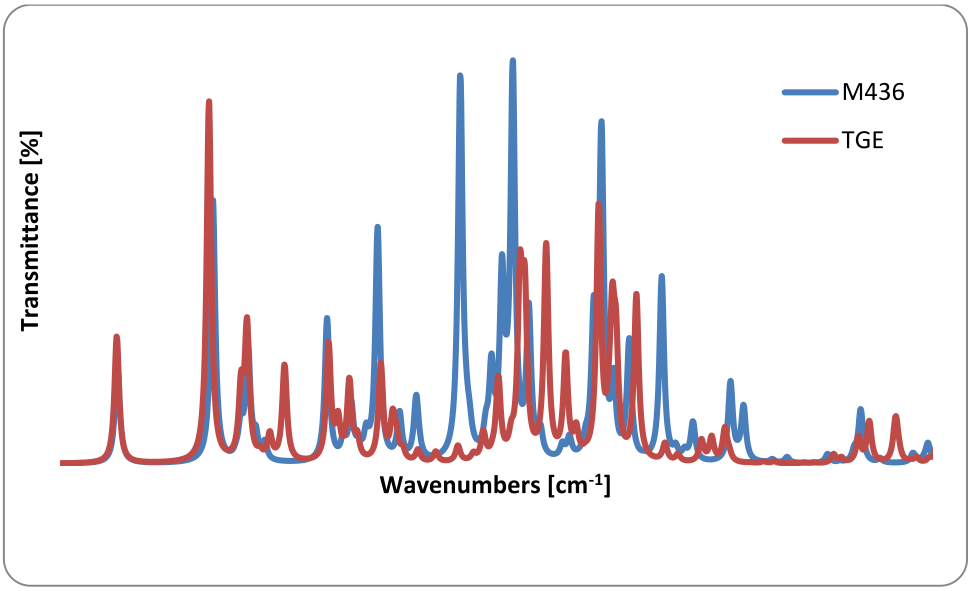

Figure S16. The comparison of theoretical IR spectra of TGE with M436.

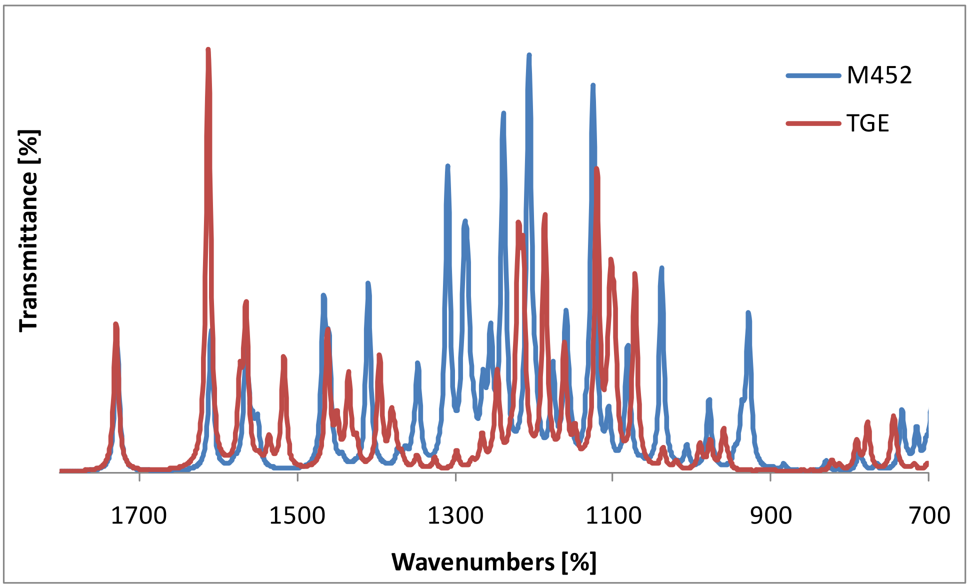

Figure S17. The comparison of theoretical IR spectra of TGE with M452.

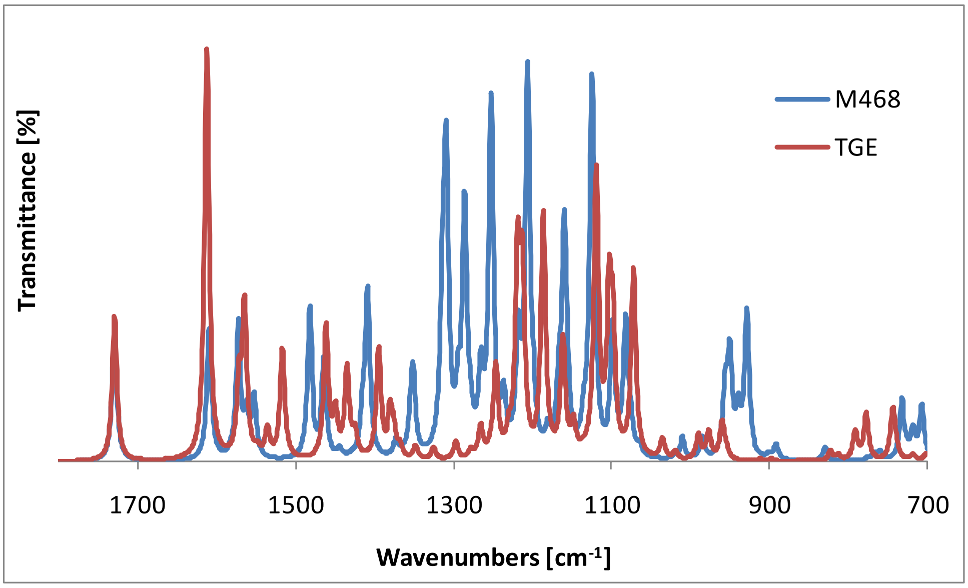

Figure S18. The comparison of theoretical IR spectra of TGE with M452.

It is known that the theoretically calculated harmonic frequencies obtained with a medium-size atomic basis sets, (e.g. with the density functional theory B3LYP/6-31G(d,p)) become overestimated with respect to the experimental IR spectra. In order to estimate at least an interesting experimental spectral region one has to reduce the theoretical values by about 10%, or to use a scaling factor, say η. Based on the available experimental spectra of the TGE and Au-TGE systems (see the experimental spectra labeled as TGE 8/098/2, ATR Di 500sk,ml and Au-TGE 2mM H_2O_2 20 min, ATR Di 1000sk, ml-2) and the theoretically calculated harmonic frequencies for the TGE and Au1-TGE molecules, we were able to estimate the value of the η-scaling factor. Details are presented in the **Table S1**.

Table S1. Comparison of the experimental IR and theoretical B3LYP/6-31G(d,p) frequencies for estimation of the η-scaling factor.

Experimental IR, in cm^{-1}	B3LYP, in cm^{-1}	Assignment	η	File name
3433	3820.14	C4′OH stretch	0.899	TGNsolo631dp
2959	3254.44	A-ring C8-H stretch	0.909	Au_1TGNdp
2927	3209.34	Intramolecular H-bond	0.912	Au_1TGNdp
2855	3137.55	CH_2CH_2 bend	0.910	Au_1TGNdp*)
2855	3168.82	C3′-H bend	0.901	Au_1TGNdp*)

*) Both theoretical frequencies are candidates to be paired with the 2855 cm^{-1} experimental IR frequency.

Taking the lowest and highest values of the η-scaling factor from the **Table S1** one can conclude that the η-values can belong to the interval < 0.899; 0.912 >.
